# Behavioural and emotional assessment of Malaysian adolescent boys with congenital colour vision deficiency and its association with socioeconomic factors

**DOI:** 10.1371/journal.pone.0353847

**Published:** 2026-07-23

**Authors:** Belina-Anne William M. Thomas, Tharenee Ramakrishnan, Sharanjeet Sharanjeet-Kaur, Mahadir Ahmad, Lei Hum Wee, Mohd Izzuddin Hairol

**Affiliations:** 1 Centre for Community Health Studies, Faculty of Health Sciences, Universiti Kebangsaan Malaysia, Kuala Lumpur, Malaysia; 2 Centre for Rehabilitation and Special Needs Studies, Universiti Kebangsaan Malaysia, Kuala Lumpur, Malaysia; 3 School of Medicine, Faculty of Health and Medical Sciences, Taylor’s University, Subang Jaya, Malaysia; Federal University of Paraiba, BRAZIL

## Abstract

Congenital colour vision deficiency (CCVD) is associated with challenges in daily functioning and social interactions, yet its behavioural and emotional implications during adolescence remain underexplored. This study aimed to compare behavioural and emotional problems between adolescent boys with CCVD and those with normal colour vision (NCV) and to examine the influence of socioeconomic factors. A school-based cross-sectional comparative study was conducted where 44 boys with CCVD and 44 age- and class-matched NCV controls (aged 13–16 years) were identified through school-based vision screening. Colour vision status was confirmed using the Ishihara and Farnsworth D15 tests. Their parents completed the Child Behaviour Checklist (CBCL/4–18) to assess *Internalising Problems, Externalising Problems*, and *Total Problems*, as well as narrow-band syndromes. Data were analysed using Mann–Whitney U tests and chi-square analyses. Adolescents with CCVD scored significantly higher on most CBCL/4–18 narrow-band scales (*Anxious/Depressed, Withdrawn, Thought Problems, Attention Problems, Delinquent Behaviour, Aggressive Behaviour*), broad-band scales *(Internalising Problems and Externalising Problems*), and *Total Problems* (all q < 0.05). *Attention Problems* showed the strongest association with CCVD status. Categorical analysis revealed that CCVD status was significantly associated with classification in the Borderline Clinical/Clinical range for *Externalising Problems, Attention Problems, Anxious/Depressed, Aggressive Behaviour*, and *Total Problems*. Among boys with CCVD, those whose parents had tertiary education demonstrated higher proportions of behavioural difficulties in the *Externalising Problems* and *Total Problems* domains; however, these findings should be interpreted with caution due to the small subgroup size. Lower household income was significantly associated with *Withdrawn, Anxious/Depressed, Thought Problems,* and *Aggressive Behaviour* scores (all q < 0.05). In conclusion, the results indicate that psychosocial and environmental factors may influence behavioural outcomes in children with CCVD, highlighting the importance of early identification and supportive interventions.

## Introduction

Individuals with congenital colour vision deficiency (CCVD) experience difficulties discriminating red and green hues. The condition is more prevalent in males than in females due to its X-linked inheritance pattern. It affects approximately 3−9% of males and 0.1–0.9% of females in most populations [1–3].

Although CCVD is a non-progressive condition, its effect on daily life experiences is considerable, which may negatively affect independence and self-esteem. In the classroom, students with CCVD also face additional challenges in tasks requiring colour discrimination, such as reading colour-coded maps, graphs, charts, and using coloured instructional materials [4,5]. Often, children with CCVD may remain unaware of their condition until they start school and face colour-dependent tasks [6].

The delay in CCVD diagnosis may impair a child’s ability to cope with CCVD, which would affect their emotional and behavioural development. Primary school children with CCVD are reported to experience more behavioural and emotional problems than their peers with normal colour vision (NCV) [7]. Specifically, these children were reported to display higher levels of aggression, withdrawal, thought-related issues, and delinquent behaviour. Unfortunately, colour vision tests are not always included in routine vision screening tests [8], which can delay CCVD diagnosis and hinder timely intervention.

Without timely intervention, these challenges may persist into adolescence, leading to more pronounced social isolation, anxiety, and depressive symptoms [4,9]. Older children with CCVD reported continued difficulties in colour-related educational and daily activities [5]. They may also be perceived as uncooperative and inattentive, resulting in frustration, embarrassment, and ridicule from both peers and teachers [10]. Furthermore, older children with CCVD who remain undiagnosed may face additional challenges when career choices requiring normal colour vision become unattainable [11,12].

We previously reported that primary school children with CCVD were perceived to be emotionally sensitive, socially withdrawn, to have a shorter attention span, and to demonstrate poorer academic performance compared to their peers with NCV [7]. However, less is known about the behavioural and emotional impact of CCVD during secondary schooling, which is characterised by increased academic demands and psychosocial challenges. Thus, the present study aimed to assess the behavioural and emotional issues and challenges faced by secondary school children with CCVD based on parental reports. These findings were compared to those of age-matched peers with NCV across ten secondary schools in Kuala Lumpur, Malaysia. Furthermore, this study explored the role of socioeconomic factors in moderating behavioural outcomes among adolescents with CCVD within a Malaysian urban school context.

## Materials and methods

### Study design and participants

This prospective, school-based cross-sectional comparative study employed purposive sampling to select participants from two groups: (1) parents of adolescent boys with CCVD as the study group, and (2) parents of adolescent boys with NCV as the control group. Adolescent boys were sampled from ten national secondary schools in Kuala Lumpur, Malaysia, randomly selected from a list of secondary schools across four districts: Kuala Lumpur (n = 4), Sentul (n = 2), Cheras (n = 2), and Sungai Besi (n = 2). These adolescent boys were identified through colour vision screening within a defined school population rather than recruited based on behavioural outcome status, consistent with a cross-sectional study design. The recruitment period was from 15 March 2018–28 April 2018.

### Sample size and power consideration

The sample size was determined based on the reported prevalence of CCVD among male schoolchildren in Selangor, Malaysia (2.6%) [13]. To achieve sufficient statistical power, we aimed to identify 40 boys with CCVD and 40 boys with NCV.

Assuming a large effect size (Cohen’s w = 0.6), an alpha level of 0.05, and 1:1 group allocation, this sample size provides approximately 80% power to detect group differences using Chi-square tests. An additional 10% was added to account for potential non-response or incomplete data, resulting in a final target of 44 participants per group (total n = 88). Screening was conducted on all eligible students in the selected schools, and recruitment was not terminated upon reaching the minimum required sample size. Adjustment for multiple comparisons using the Benjamini–Hochberg False Discovery Rate (FDR) procedure was applied during analysis and was not incorporated into the a priori sample size estimation.

### Recruitment and consent

A description of the study and consent forms were distributed to all boys aged 13–16 years in the selected secondary schools to be handed to their parents. The age group was selected to enable the assessment of behavioural and emotional difficulties during the secondary school years, a developmental period associated with increasing academic and social demands. Moreover, adolescents in this age range are likely cognitively mature enough to comprehend their colour vision status, which may support informed educational and future career decisions.

Boys whose parents provided written consent were invited to participate in a vision screening session held at the school. Parents were also asked to complete a socio-demographic questionnaire, which collected information on parental age, gender, ethnicity, marital status, education level, monthly household income, and awareness of their child’s colour vision difficulties. The parents were also required to report their child’s medical conditions (including psychological or psychiatric consultations), ocular history, and current medications. Only adolescent boys who gave verbal assent and obtained parental written consent underwent vision screening.

### Vision screening

All eligible adolescent boys underwent visual acuity testing using the Early Treatment Diabetic Retinopathy Study (EDTRS) chart. Only those with visual acuity of at least 0.2 logMAR (Snellen 6/9) with best correction proceeded to colour vision screening. Those with self-reported monocular and/or binocular vision disorders, systemic health problems, eye disease, and psychological conditions were excluded.

### Colour vision assessment

Colour vision screening was conducted using the Ishihara Pseudoisochromatic 24-Plate Test. The test was performed under the room illumination of 350 lux, consistent with clinical standards [14]. The plates were presented at a viewing distance of 75 cm perpendicular to the line of sight and viewed binocularly. Each plate was presented for up to four seconds, and the number of identification errors was recorded. The adolescent boys were classified as failing the test if they made four or more errors.

Those who failed the Ishihara Test then performed the Farnsworth D15 test to determine the type of colour vision deficiency. The test comprised 15 movable coloured caps and one fixed reference coloured cap. After the 15 caps were arranged randomly on a black surface, participants were instructed to place them in sequential order of hue, starting from the reference cap. Participants were allowed to alter the cap arrangement before confirming their final sequence. Testing was conducted binocularly at approximately 50 cm under Illuminant C lighting (270 lux), and the allocated time was two minutes. The test was first done to ensure that participants understood the procedure, and then it was repeated for recording.

The arrangement of the caps was recorded. Errors were scored using the sequence numbers printed on the reverse side of each cap. A diagrammatic plot of the sequence was used to identify crossing patterns relative to protan, deutan, and tritan axes.

The presence and type of CCVD were confirmed depending on the crossings obtained. If the lines remained outside the circle or there were minor errors (for example, reversing the order of adjacent caps or one to two positions from the correct arrangement), the participants were said to be ‘normal’ or have a mild colour defect.

Major errors, defined as two or more diametrical crossings, were widely accepted as the best criterion for failure, indicating a medium or severe defect. The type of defect was determined by comparing these crossover lines to see if they paralleled the protan, deutan or tritan colour confusion axes. Results were confirmed using a web-based scoring software (https://www.torok.info/colorvision/d15.thm).

Once the confirmation was made, the adolescent boys were grouped into the CCVD group, and their parents were identified. Then, parents of an equal number of adolescent boys with NCV (age- and class-matched) were selected as controls.

### Behavioural and emotional assessment

The Child Behaviour Checklist (CBCL/4–18) [15], a parent-report questionnaire, was used to evaluate behavioural and emotional problems in adolescents. The Malay language version was used, which has been validated in prior studies involving Malay-speaking populations [7,16]. The CBCL/4–18 comprises 113 problem items rated on a 3-point Likert scale (0 = not true, 1 = somewhat or sometimes true, 2 = very true or often true) based on behaviours observed over the past six months.

The CBCL provides scores for syndrome scales, *Internalising Problems* (e.g., anxiety, depression, withdrawal), *Externalising Problems* (e.g., aggression, rule-breaking), and a *Total Problems* score. Raw scores were converted to T-scores using age- and gender-specific norms, categorising responses into normal, borderline clinical, and clinical ranges. A T-score >63 on broadband scales or >70 on narrowband scales was considered clinically significant. T-scores of 60–63 and 67–70 were classified as borderline for broadband and narrowband scales, respectively. Higher scores in these ranges indicated increased severity and the need for further evaluation. The CBCL has demonstrated high internal consistency (α = 0.72–0.96) and strong validity across populations [15–17].

The CBCL was distributed individually to parents of the identified adolescents through the participating schools. Parents were provided with written instructions explaining how to complete the questionnaire and were encouraged to contact the research team if clarification was required. No group demonstration sessions were conducted to minimise response bias. Completed questionnaires were returned to the school within one week of distribution.

### Statistical analysis

Data were analysed using IBM SPSS Statistics version 23. Normality of data distribution was assessed using the Shapiro–Wilk test, as it is recommended for small samples (<50 per group) [18,19]. Descriptive statistics were generated, followed by appropriate parametric or non-parametric tests to compare behavioural and emotional outcomes between the CCVD and NCV groups.

Participants’ CBCL/4–18 T-scores were classified into two categories (normal and combined Borderline Clinical and Clinical). Associations between participant group (CCVD vs. NCV) and T-score category for each CBCL/4–18 scale were examined using Pearson’s Chi-square test of independence. Effect sizes for the Chi-square analyses were reported using Cramér’s V, along with 95% confidence intervals for Cramér’s V, which were estimated using non-parametric bootstrapping (1,000 resamples, percentile method). Within the CCVD group, additional Chi-square tests were performed to examine associations between T-score category (Normal vs. combined Borderline Clinical/Clinical) and parental/familial socio-demographic variables, which included parental education level (secondary vs. tertiary) and monthly household income (low vs. high), and parental awareness of their child’s CCVD status (aware vs. unaware). The household income cut-off of MYR 4,850 was prespecified based on national income benchmarks to differentiate lower- and higher-income groups.

Given the interrelated nature of CBCL/4–18 scales, adjustment for multiple comparisons was performed using the Benjamini–Hochberg False Discovery Rate (FDR) method. Both unadjusted p-values and FDR-adjusted q-values are reported. Statistical inference was based on q-values, with statistical significance defined as q < 0.05.

### Ethical considerations

This study was conducted in accordance with the tenets of the Declaration of Helsinki and approved by the Research Ethics Committee, Universiti Kebangsaan Malaysia (UKM PPI/111/8/JEP-2018–124), the Ministry of Education Malaysia (MOE) (Reference Num.: KPM.600–3/2/3 Vol. 58(7)) and the Department of Education, Federal Territory of Kuala Lumpur (Reference Num.: JPNWP.900–6/1/7 Jld. 26(68)).

## Results

Of the 2,879 adolescent boys who underwent the vision screening, 44 (1.53%) were identified as having CCVD, and all of them were deutans. All identified adolescents with CCVD and age-matched peers with normal colour vision were aged 13–16 years (mean age = 14.52 ± 1.30 years), enrolled in Forms 1–4 of secondary school, and were of Malay ethnicity.

Eighty-eight parents participated in the study, consisting of 44 parents of boys with CCVD and 44 parents of boys with NCV. Only 12 out of 44 mothers of boys with CCVD (27.3%) were aware of their son’s colour-related difficulties, based on parental observations of colour-related difficulties and/or a known family history of colour vision deficiency, rather than prior formal diagnosis by an eye care professional. Table 1 summarises the sociodemographic characteristics of the participants.

**Table 1:** Socio-demographic characteristics of the participants.

Variable	CCVD group (n = 44)	NCV group (n = 44)
Parent age (range), n (%)		
31–50 years	26 (59.1%)	22 (50.0%)
> 51 years	18 (40.9%)	22 (50.0%)
Marital status, n (%)		
Single parent	2 (4.5%)	0 (0.0%)
Married	42 (95.5%)	44 (100.0%)
Education level, n (%)		
Secondary	37 (84.1%)	35 (79.5%)
Tertiary	7 (15.9%)	9 (20.5%)
Family income (range), n (%)		
Low (≤MYR4850)	13 (29.5%)	12 (27.3%)
High (>MYR4850)	31 (70.5%)	32 (72.7%)
Awareness of CCVD, n (%)	12 (27.3%)	–

Adolescent boys with CCVD had higher mean scores than those with NCV across all eight narrow-band scales, except for *Somatic Complaints*. Similarly, higher mean scores were observed in the CCVD group for the two broad-band scales *(Internalising Problems* and *Externalising Problems*) and for *Total Problems* (Table 2).

**Table 2:** CBCL/4-18 scale scores by group (mean and standard deviation).

CBCL/4–18 scale (mean±sd)	CCVD (n = 44)	NCV (n = 44)
**Narrow band**		
Withdrawn	62.18 ± 9.794	55.70 ± 6.667
Somatic complaints	57.14 ± 7.020	59.93 ± 8.932
Anxious/Depressed	56.18 ± 5.780	52.52 ± 5.133
Social problems	59.16 ± 9.433	57.18 ± 7.500
Thought problems	59.48 ± 8.951	54.39 ± 5.954
Attention problems	60.77 ± 12.447	53.27 ± 5.169
Delinquent behaviour	55.84 ± 4.822	51.77 ± 4.513
Aggressive behaviour	56.48 ± 8.048	52.18 ± 2.871
**Broad band**		
Internalising problems	56.75 ± 11.437	53.48 ± 9.735
Externalising problems	52.34 ± 11.986	46.25 ± 9.984
**Total behaviour problems**		
Total problems	57.70 ± 12.285	51.73 ± 9.217

Normality testing with the Shapiro-Wilk test indicated that all mean scores were not normally distributed (p < 0.05 for all scales). Therefore, the Mann-Whitney U test was applied for group comparisons. Statistical significance was determined using FDR-adjusted q-values, with q < 0.05 considered statistically significant, as described in the Methods.

Significant differences were found for both broad-band scales, where adolescent boys with CCVD scored significantly higher on *Internalising Problems* and *Externalising Problems* compared with those in the NCV group (both q < 0.05). Among the narrow-band scales, the CCVD group scored significantly higher on six scales: *Withdrawn, Anxious/Depressed, Thought Problems, Attention Problems, Delinquent Behaviour,* and *Aggressive Behaviour* (all q < 0.05). In contrast, the NCV scored significantly higher for *Somatic Complaints* (U = 712.50, q = 0.034), while no significant difference was observed for *Social Problems* (U = 951.00, q = 0.88). Additionally, adolescent boys with CCVD scored significantly higher for *Total Problems* than the NCV group (U = 600.00, q = 0.002). Table 3 summarises the findings.

**Table 3:** CBCL/4-18 scale scores by group (mean rank).

CBCL/4–18 scale (mean rank)	CCVD (n = 44)	NCV (n = 44)	U	p-value	q-value
**Narrow band**					
Withdrawn	52.86	36.14	600.00	0.002*	0.005*
Somatic complaints	38.69	50.31	712.50	0.03*	0.034*
Anxious/Depressed	54.76	34.24	516.50	<0.001*	0.005*
Social problems	44.89	44.11	951.00	0.88	0.88
Thought problems	52.10	36.90	633.50	0.003*	0.006*
Attention problems	51.36	37.64	666.00	0.01*	0.016*
Delinquent behaviour	55.80	33.20	471.00	<0.001*	0.005*
Aggressive behaviour	50.86	38.32	696.00	0.02*	0.027*
**Broad band**					
Internalising problems	50.48	38.52	705.00	0.03*	0.03*
Externalising problems	51.57	37.43	657.00	0.009*	0.018*
**Total behaviour problems**					
Total problems	52.86	36.14	600.00	0.002*	0.002*

*Both unadjusted p-values and FDR-adjusted q-values are reported; statistical significance was defined as q < 0.05.

Pearson’s Chi-square tests of independence were performed to examine the association between adolescent boys’ group (CCVD vs. NCV) and T-score category (Normal vs. combined Borderline Clinical/Clinical) across CBCL scales (Table 4). There was a statistically significant association between adolescent boys’ group and T-score category for the *Anxious/Depressed* scale, χ^²^(1, N = 44) = 7.605, p = 0.006, q = 0.012, Cramér’s V = 0.294, 95% CI [0.167, 0.405], *Attention Problems* scale χ^²^(1, N = 44) = 16.65, p < 0.001, q = 0.005, Cramér’s V = 0.435, 95% CI [0.316, 0.562], *Aggressive Behaviour* scale χ^²^(1, N = 44) = 7.61, p = 0.006, q = 0.012, Cramér’s V = 0.294, 95% CI [0.167, 0.405], *Externalising Problems*, χ^²^(1, N = 44) = 15.253, p < 0.001, q = 0.002, Cramér’s V = 0.416, 95% CI [0.301, 0.537] and *Total Problems*, χ^²^(1, N = 44) =10.700, p = 0.001, q = 0.001, Cramér’s V = 0.349, 95% CI [0.153, 0.537].

**Table 4:** Associations between participant group and T-score category for each CBCL/4-18 scale.

CBCL/4–18 Scale	Group	T-Score category, n (%)		χ^2^	p-value	q-value	Cramér’s V, 95% CI
		Normal	Borderline clinical/Clinical				
**Narrow-band**							
Withdrawn	CCVD	31 (70.50%)	13 (29.50%)	3.289	0.07	0.078	0.193 [0.017, 0.394]
	NCV	38 (86.40%)	6 (13.60%)				
Anxious/Depressed	CCVD	37 (84.10%)	7 (15.90%)	7.605	0.006	0.012*	0.294 [0.167, 0.405]
	NCV	44 (100%)	0 (0%)				
Thought Problems	CCVD	31 (70.50%)	13 (29.50%)	3.289	0.07	0.078	0.193 [0.017, 0.394]
	NCV	38 (86.40%)	6 (13.60%)				
Attention Problems	CCVD	30 (68.20%)	14 (31.80%)	16.649	<0.001	0.005*	0.435 [0.316, 0.562]
	NCV	44 (100%)	0 (0%)				
Delinquent Behaviour	CCVD	44 (100%)	0 (0%)	–	–	–	–
	NCV	44 (100%)	0 (0%)				
Aggressive Behaviour	CCVD	37 (84.10%)	7 (15.90%)	7.605	0.006	0.012*	0.294 [0.167, 0.405]
	NCV	44 (100%)	0 (0%)				
**Broad-band**							
Internalising Problems	CCVD	18 (40.90%)	26 (59.10%)	2.228	0.135	0.135	0.159 [0.008, 0.364]
	NCV	25 (56.80%)	19 (43.20%)				
Externalising Problems	CCVD	31 (70.50%)	13 (29.50%)	15.253	<0.001	0.002*	0.416 [0.301, 0.537]
	NCV	44 (100%)	0 (0%)				
**Total Problem**							
Total Problems	CCVD	24 (54.50%)	20 (45.50%)	10.700	0.001	0.001*	0.349 [0.153, 0.537]
	NCV	38 (86.40%)	6 (13.60%)				

Among adolescent boys with CCVD, there were statistically significant relationships between their parental education level and behavioural outcomes on broad-band CBCL scales (Table 5); specifically for broad-band scale: *Externalising Problems,* χ^²^(1, N = 44) = 12.617, p < 0.001, q = 0.002, Cramér’s V = 0.535, 95% CI [0.220, 0.787] and *Total Problems,* χ^²^(1, N = 44) = 5.442, q = 0.02, Cramér’s V = 0.352, 95% CI [0.066, 0.594] that showed strong to moderate effect sizes. These results indicate a significant relationship between parental education and behavioural outcomes among adolescents with CCVD. For *Externalising Problems*, seven adolescents whose parents had secondary education and six adolescents whose parents had tertiary education were classified within the borderline/clinical range. However, proportionally, the prevalence was higher in the tertiary education group (85.7%) than the secondary education group (18.9%) (p < 0.001). Similarly, for *Total Problems*, 14 adolescents whose parents had secondary education and six adolescents whose parents had tertiary education were classified within the borderline/clinical range, with a higher proportion observed among the tertiary education group (85.7%) than the secondary education group (37.8%).

**Table 5:** Associations between adolescent boys with CCVD’s parental education level and T-score category for each CBCL/4-18 scale.

CBCL/4–18 Scale	Parental Education Level	T-Score category, n (%)		χ^2^	p-value	q-value	Cramér’s V, 95% CI
		Normal	Borderline clinical/Clinical				
**Narrow-band**							
Withdrawn	Secondary	24 (64.90%)	13 (35.10%)	3.491	0.062	0.124	0.282 [0.156, 0.411]
	Tertiary	7 (100.00%)	0 (0%)				
Anxious/Depressed	Secondary	30 (81.10%)	7 (18.90%)	1.575	0.210	0.233	0.189 [0.097, 0.293]
	Tertiary	7 (100.00%)	0 (0%)				
Thought Problems	Secondary	24 (64.90%)	13 (35.10%)	3.491	0.062	0.124	0.282 [0.156, 0.411]
	Tertiary	7 (100.00%)	0 (0%)				
Attention Problems	Secondary	23 (62.20%)	14 (37.80%)	3.885	0.049	0.245	0.297 [0.173, 0.440]
	Tertiary	7 (100.00%)	0 (0%)				
Delinquent Behaviour	Secondary	37 (100%)	0 (0%)	–	–		–
	Tertiary	7 (100%)	0 (0%)				
Aggressive Behaviour	Secondary	30 (81.10%)	7 (18.9%)	1.575	0.210	0.233	0.189 [0.097, 0.293]
	Tertiary	7 (100%)	0 (0%)				
**Broad-band**							
Internalising Problems	Secondary	17 (45.90%)	20 (54.10%)	2.441	0.118	0.118	0.236 [0.021, 0.435]
	Tertiary	1 (14.30%)	6 (85.70%)				
Externalising Problems	Secondary	30 (81.10%)	7 (18.90%)	12.617	<0.001	0.002*	0.535 [0.220, 0.787]
	Tertiary	1 (14.30%)	6 (85.70%)				
**Total Problem**							
Total Problems	Secondary	23 (62.20%)	14 (37.80%)	5.442	0.02	0.02*	0.352 [0.066, 0.594]
	Tertiary	1 (14.30%)	6 (85.70%)				

Significant associations were also identified between monthly household income (low vs. high) and behavioural problems among adolescents with CCVD (Table 6). Narrow-band scales showing significant associations included *Withdrawn,* χ^²^(1, N = 44) = 26.882, q = 0.002, Cramér’s V = 0.782, 95% CI [0.546, 0.951], *Anxious/Depressed,* χ^²^(1, N = 44) = 12.617, q = 0.002, Cramér’s V = 0.535, 95% CI [0.239, 0.795], *Thought Problems,* χ^²^(1, N = 44) = 26.882, q = 0.002, Cramér’s V = 0.782, 95% CI [0.546, 0.951], and *Aggressive Behaviour,* χ^²^(1, N = 44) = 12.617, q = 0.002, Cramér’s V = 0.535, 95% CI [0.239, 0.795]. In other words, there is a significant relationship between lower monthly household income and some behavioural difficulties among adolescents with CCVD.

**Table 6:** Associations between adolescent boys with CCVD’s parental monthly household income and T-score category for each CBCL/4-18 scale.

CBCL/4–18 Scale	Parental Monthly Household Income	T-Score category, n (%)		χ^2^	p-value	q-value	Cramér’s V, 95% CI
		Normal	Borderline clinical/Clinical				
**Narrow-band**							
Withdrawn	Low	2 (15.40%)	11 (84.60%)	26.882	<0.001	0.002*	0.782 [0.546, 0.951]
	High	29 (93.50%)	2 (6.50%)				
Anxious/Depressed	Low	7 (53.80%)	6 (46.20%)	12.617	<0.001	0.002*	0.535 [0.239, 0.795]
	High	30 (96.80%)	1 (3.20%)				
Thought Problems	Low	2 (15.40%)	11 (84.60%)	26.882	<0.001	0.002*	0.782 [0.546, 0.951]
	High	29 (93.50%)	2 (6.50%)				
Attention Problems	Low	7 (53.80%)	6 (46.20%)	1.748	0.186	0.186	0.199 [0.010, 0.520]
	High	23 (74.20%)	8 (25.80%)				
Delinquent Behaviour	Low	13 (100%)	0 (0%)	–	–	–	–
	High	31 (100%)	0 (0%)				
Aggressive Behaviour	Low	7 (53.80%)	6 (46.20%)	12.617	<0.001	0.002*	0.535 [0.239, 0.795]
	High	30 (96.80%)	1 (3.20%)				
**Broad-band**							
Internalising Problems	Low	7 (53.80%)	6 (46.20%)	1.278	0.258	0.258	0.170 [0.009, 0.474]
	High	11 (35.50%)	20 (64.50%)				
Externalising Problems	Low	7 (53.80%)	6 (46.20%)	2.445	0.118	0.236	0.236 [0.017, 0.546]
	High	24 (77.40%)	7 (22.60%)				
**Total Problem**							
Total Problems	Low	7 (53.80%)	6 (46.20%)	0.004	0.952	0.952	0.009 [0.002, 0.336]
	High	17 (54.80%)	14 (45.20%)				

Within the CCVD group, parental awareness of their child’s CCVD status (unaware vs. aware) was significantly associated with selected behavioural problems on the CBCL/ 4–18 narrow-band scales (Table 7). Adolescents whose parents were unaware of their CCVD status were more likely to score within the borderline clinical/clinical range for *Withdrawn,* χ^²^(1, N = 44) = 6.919, q = 0.018, Cramér’s V = 0.397, 95% CI [0.266, 0.544], *Thought Problems,* χ^²^(1, N = 44) = 6.919, q = 0.018, Cramér’s V = 0.397, 95% CI [0.266, 0.544], and *Attention Problems,* χ^²^(1, N = 44) = 7.700, q = 0.03, Cramér’s V = 0.418, 95% CI [0.272, 0.585]. Notably, all parents who were aware of their child’s CCVD status (n = 12) rated their adolescents within the normal range across all CBCL/4–18 narrow-band scales, whereas elevated scores were observed exclusively among adolescents whose parents were unaware of the condition. No statistically significant associations were observed between parental awareness and broad-band scales (*Internalising Problems*, *Externalising Problems*, and *Total Problems*) (all q > 0.05).

**Table 7:** Associations between adolescent boys with CCVD’s parental awareness of their child’s CCVD status and T-score category for each CBCL/4-18 scale.

CBCL/4–18 Scale	Parental Awareness of their Child’s CCVD Status	T-Score category, n (%)		χ^2^	p-value	q-value	Cramér’s V, 95% CI
		Normal	Borderline clinical/Clinical				
**Narrow-band**							
Withdrawn	Unaware	19 (59.40%)	13 (40.60%)	6.919	0.009	0.018*	0.397 [0.266, 0.544]
	Aware	12 (100%)	0 (0%)				
Anxious/Depressed	Unaware	25 (78.10%)	7 (21.90%)	3.122	0.077	0.086	0.266 [0.156, 0.394]
	Aware	12 (100%)	0 (0%)				
Thought Problems	Unaware	19 (59.40%)	13 (40.60%)	6.919	0.009	0.018*	0.397 [0.266, 0.544]
	Aware	12 (100%)	0 (0%)				
Attention Problems	Unaware	18 (56.30%)	14 (25.80%)	7.700	0.006	0.03*	0.418 [0.272, 0.585]
	Aware	12 (100%)	0 (0%)				
Delinquent Behaviour	Unaware	32 (100%)	0 (0%)	–	–	–	–
	Aware	12 (100%)	0 (0%)				
Aggressive Behaviour	Unaware	25 (78.10%)	7 (21.90%)	3.122	0.077	0.086	0.266 [0.156, 0.394]
	Aware	12 (100%)	0 (0%)				
**Broad-band**							
Internalising Problems	Unaware	12 (37.50%)	20 (62.50%)	0.564	0.453	0.453	0.113 [0.005, 0.419]
	Aware	6 (50.00%)	6 (50.00%)				
Externalising Problems	Unaware	25 (78.10%)	7 (21.90%)	3.316	0.069	0.138	0.275 [0.017, 0.564]
	Aware	6 (50.00%)	6 (50.00%)				
**Total Problem**							
Total Problems	Unaware	18 (56.30%)	14 (25.80%)	0.138	0.711	0.711	0.056 [0.003, 0.354]
	Aware	6 (50.00%)	6 (50.00%)				

## Discussion

The present study revealed that adolescent boys with CCVD exhibited significantly higher scores on several CBCL/4–18 narrow-band scales, including *Anxious/Depressed, Withdrawn, Thought Problems, Attention Problems, Delinquent Behaviour*, and *Aggressive Behaviour*. Among these, the strongest associations with CCVD status were observed for *Attention Problems*, followed by *Externalising Problems* and *Total Problems*. The findings suggest that adolescent boys with CCVD may experience a broader pattern of emotional and behavioural difficulties compared with their peers with normal colour vision. The strongest association between CCVD and Attention Problems suggests that attentional and behavioural regulation difficulties may represent particularly important challenges in this population. The concurrent elevation in *Internalising Problems, Externalising Problems,* and *Total Problems* further indicates that CCVD is associated with broader psychosocial and behavioural outcomes beyond colour-related functional limitations. Overall, these results support the possibility that CCVD is associated with increased vulnerability to emotional, attentional, and behavioural difficulties during adolescence. These findings are consistent with previous reports documenting psychosocial and emotional challenges faced by individuals with CCVD [7,20] and further support an association between CCVD and increased psychosocial and emotional difficulties during development.

Behavioural and emotional functioning during adolescence may be influenced by developmental and pubertal changes [21,22]. However, in the present study, adolescent boys with CCVD were compared with age-matched peers with NCV, thereby minimising the potential confounding effects of pubertal development. Accordingly, while age-related factors may have influenced CBCL scores overall, the study’s findings suggest that the elevated behavioural and emotional problem scores observed among adolescent boys with CCVD are unlikely to be attributable solely to normal adolescent developmental or pubertal changes. Because participants were compared with age-matched peers with normal colour vision, the observed group differences more likely reflect factors associated with CCVD itself rather than general age-related developmental influences. This further supports an association between CCVD and increased emotional and behavioural vulnerability during adolescence.

An important finding of this study was that elevated behavioural scores were observed exclusively among adolescents whose parents were unaware of their CCVD status, whereas all adolescents whose parents were aware of the children’s CCVD status were rated within the normal range across narrow-band scales. This pattern suggests that parental awareness, even in the absence of a formal clinical diagnosis, may play an important role in shaping how behavioural difficulties are perceived and interpreted. When parents recognise the functional limitations associated with CCVD, academic or attentional challenges may be more accurately attributed to visual constraints rather than misinterpreted as behavioural or emotional problems. Alternatively, the observed differences may reflect variation in parental perception or reporting, rather than true differences in symptom severity, particularly given the reliance on parent-reported measures in this study. However, given the relatively small subgroup sample size and multiple comparisons examined, these findings should be interpreted as exploratory and hypothesis-generating, and future studies with larger samples are needed to confirm these associations.

Children and adolescents with CCVD may struggle to understand and communicate their difficulties to others, often resulting in frustration and withdrawal [23]. When such difficulties are misunderstood or minimised by adults and peers, adolescents may internalise these experiences as personal inadequacies. In line with these observations, the present study found significantly elevated scores in *Anxious/Depressed* and *Withdrawn* scales among adolescents with CCVD, underscoring the role of emotional vulnerability during a developmental period characterised by increasing academic complexity, heightened social expectations, and greater sensitivity to peer evaluation.

In line with these findings, literature highlights the broader behavioural and emotional impact of CCVD across childhood and adolescence. Although children with CCVD often appear developmentally typical, repeated difficulties with colour-dependent academic tasks can lead to frustration, embarrassment, and diminished self-confidence when these challenges are misunderstood by adults or peers [4,24,25]. Beyond academics, difficulties in sports and play, including distinguishing team colours or tracking objects against similarly coloured backgrounds, may further impair peer interactions and social participation [4]. Over time, such repeated experiences of failure and misunderstanding may contribute to internalising behaviours, withdrawal, and externalising responses, particularly during adolescence when academic demands and social expectations intensify. Reviews and commentaries further suggest that delayed identification of CCVD may negatively affect well-being, self-esteem, and future educational or career choices, with late diagnosis sometimes triggering psychological distress, anger, or grief [6,20].

The present study’s results also build on an earlier work [7], which reported that primary school children with CCVD scored significantly higher on CBCL/4–18’s narrow-band scales such as *Withdrawn, Thought Problems*, and *Aggressive Behaviour*, as well as on *Externalising Problems* and *Total Problems*. While both studies indicate a significant association between CCVD status and increased behavioural and emotional difficulties, the adolescent sample in the current study exhibited a broader and more pronounced spectrum of issues, including significant elevations in *Anxious/Depressed* and *Attention Problems* scales, which were less prominent in younger children with CCVD. The pattern suggests a possible progression in the psychosocial impact of CCVD in children with age, likely influenced by increasing academic complexity and social demands during adolescence [10,26,27]. As older children with CCVD become more aware of their visual limitations, they may experience greater frustration and anxiety, particularly in environments that rely heavily on colour-coded tasks and in peer interactions [10,27]. Thus, having CCVD may be associated with psychological stress and negatively impact self-esteem, classroom engagement, and social withdrawal among adolescents [5].

Importantly, the present study also observed that these behavioural and emotional difficulties are further amplified among adolescents with CCVD from lower socioeconomic backgrounds. The present study found that adolescents with CCVD from lower-income households demonstrated substantially higher proportions of behavioural problems like *Withdrawn*, *Anxious/Depressed*, *Thought Problems* and *Aggressive Behaviour*. This finding is consistent with previous literature suggesting that financial constraints may contribute to psychosocial stressors, reduced access to support resources, and increased emotional and behavioural difficulties among children and adolescents [28]. Similarly, a Malaysian study also demonstrated that family socioeconomic status significantly influences young Malaysian children’s social-emotional development, with lower socioeconomic status linked to poorer outcomes [29]. Our study extends these findings by demonstrating that low socioeconomic status may exacerbate the psychosocial impact of often overlooked conditions such as CCVD. Adolescents with CCVD face additional stressors, such as limited access to diagnosis and insufficient support [10,27]. Low socioeconomic status is associated with worsened emotional and behavioural outcomes, which may not be as apparent in adolescents with CCVD from higher socioeconomic status groups. This underscores the importance of socioeconomic context in shaping the behavioural and emotional outcomes among adolescents with CCVD and highlights the need for targeted interventions that account for these disparities. In contrast, higher proportions of *Externalising Problems* and *Total Problems* were observed among adolescents whose parents had tertiary education attainment. Given the small sample size (n = 7) within the tertiary education subgroup, this finding should be interpreted cautiously and may reflect sampling imbalance or differences in parental awareness and reporting behaviours. In addition, parents with higher educational attainment may be more aware of behavioural and emotional concerns, potentially contributing to increased reporting of such difficulties.

Given these observations, early screening, diagnosis, and targeted interventions for CCVD are crucial. Vision and eye health screening programs play a crucial role in identifying students with vision disorders, as undiagnosed conditions are often associated with higher rates of internalising behaviours (such as anxiety and withdrawal) and externalising behaviours (such as aggression) [30,31]. Research shows that early identification of vision problems and timely intervention and support may help alleviate academic challenges and reduce associated emotional or behavioural issues [32,33]. Despite CCVD’s high prevalence among males (3–8% globally) [1–3], colour vision screening is often absent from school health programs [8]. Thus, incorporating routine screening for CCVD in schools would facilitate timely academic accommodations and psychological support, reducing the risk of academic frustration, social isolation, and emotional distress. The low level of parental awareness of their child’s CCVD status could be due to the lack of routine school-based colour vision screening and access to formal diagnosis among school-aged children. Thus, raising awareness among parents and educators about vision problems in adolescents, including CCVD, may help improve understanding, reduce stigma, foster a supportive learning environment and increase social participation for affected adolescents [34–36].

The key strength of the current study lies in its design, particularly the pairwise matching of adolescents by age, school, and class. However, several limitations need to be considered. First, the sample was restricted to boys, reflecting the higher prevalence of CCVD among males. Future studies should examine whether similar behavioural and emotional patterns are observed among adolescent girls with CCVD. Second, pubertal status and hormonal influences, which may affect behavioural and emotional regulation during adolescence, were not directly assessed. Although age-matching was used to minimise developmental confounding, residual variability related to pubertal development cannot be entirely excluded and may have contributed to differences in CBCL/4–18 scores. Third, behavioural functioning was assessed solely using parent-reported CBCL/4–18 measures, which may be subject to reporting bias or under- or over-estimation of symptoms. The inclusion of adolescent self-reports, teacher reports, or structured mental health assessments in future studies would provide a more comprehensive evaluation of behavioural and emotional functioning. Fourth, parental awareness of CCVD was based on observation or family history rather than prior clinical diagnosis, which may have influenced parental ratings. In addition, the small number of parents reporting awareness limited the ability to conduct subgroup analyses. Future studies incorporating clinically confirmed diagnoses and multi-informant assessments would strengthen the interpretation of these findings. Fifth, the Farnsworth D-15 test used in this study primarily detects moderate to severe CCVD, which may have resulted in under-identification of adolescents with mild colour vision deficiencies. In addition, all CCVD cases identified in the present study were classified as deutan defects, which may limit the generalisability of the findings to other forms of congenital colour vision deficiency, particularly protan defects.

## Conclusion

Adolescent boys with CCVD were reported to have significantly higher behavioural and emotional problems compared to peers with NCV. Elevated scores were observed on multiple CBCL/4–18 narrow-band scales, particularly *Anxious/Depressed*, *Withdrawn*, *Thought Problems*, *Attention Problems*, *Delinquent Behaviour*, and *Aggressive Behaviour*, as well as on broad-band scales (*Internalising Problems and Externalising Problems*) and *Total Problems*. They are also more likely to fall within the Borderline Clinical/Clinical range for *Externalising Problems*, *Attention Problems*, *Anxious/Depressed*, *Aggressive Behaviour*, and *Total Problems*. Adolescent boys from lower-income households demonstrated substantially higher proportions of behavioural problems like *Withdrawn, Anxious/Depressed, Thought Problems* and *Aggressive Behaviour*. In contrast, higher proportions of *Externalising Problems* and *Total Problems* were observed among adolescents whose parents had tertiary education attainment. Given the small sample size within the tertiary education subgroup, this finding should be interpreted cautiously and may reflect sampling imbalance or differences in parental awareness and reporting behaviours. Routine school-based colour vision screening, coupled with early behavioural and emotional assessment and targeted educational support, may help support adolescents and reduce potential long-term psychosocial challenges among adolescents with CCVD.
