# Supplementary material for: Behavioural and emotional assessment of Malaysian adolescent boys with congenital colour vision deficiency and its association with socioeconomic factors
Source: PLoS One. 2026 Jul 23;21(7):e0353847. doi: 10.1371/journal.pone.0353847 (PMC13395452; doi:10.1371/journal.pone.0353847)
Supplement: Attachment — Submitted filename: PLOS Review on CCVD.docx [file pone.0353847.s001.docx]

**Behavioural and emotional issues in Malaysian adolescent boys with congenital colour vision deficiency and their association with socioeconomic factors**

**Title:**

The authors used a behavioral checklist, so the title should reflect the behavioral assessment of Malaysian adolescent boys with congenital color vision deficiency and its association with socio-demographic factors.

**Abstract:**

1. You have not assessed academic performance in your study. Therefore, it may be misleading to state at the beginning that ‘Congenital colour vision deficiency (CCVD) can impact academic performance and social interactions”.
2. Why was this not conducted as a case-control study? Please justify your choice of study design.

**Introduction**

Line 32: You have not mentioned any comment regarding socio-economic factors in the objectives section of the introduction. “Thus, the present  study aimed to assess the behavioural and emotional issues and challenges faced by secondary school children with CCVD based on parental reports. These findings were compared to those of age-matched peers with NCV across ten secondary schools in Kuala Lumpur, Malaysia.”

**Materials and methods**

**Study design and participants**

Why was this not conducted as a case-control study? Please justify your choice of study design.

**Behavioral and emotional assessment:**

Line 112: it is mentioned that “Parents of the identified adolescents completed the CBCL questionnaire and returned it within one week of distribution”. Did you distribute all the forms in a parents’ assembly on the same day or were they distributed individually? Did you demonstrate to them how to fill out the form? Please describe this process clearly in the methodology section.

**Statistical analysis:**

Please mention whether any adjustment was made to determine the significant p-value from the interrelated scores.

**Result:**

1. Line 137: “Of the 2,879 adolescent boys who underwent vision screening, 44 (1.53%) were identified as having CCVD, and all of them were deutans.” Which classes were they in, and how old were they? All of them were between 11 and 13 years of age? Is If yes, was there any specific reason for choosing the age group 11–13 years?Please provide a suitable explanation.
2. You mentioned that 40 samples are needed in each group to maintain adequate statistical power. In the results section, you reported 44 boys with congenital colour vision defect. Was this coincidental, or did you stop screening once you reached the desired sample size? Please provide a suitable explanation in the methodology or results section.
3. No need to mention gender of mother in the table 1.
4. Line 143: Only 12 out of 44 mothers of boys with CCVD (27.3%) were aware of their son’s CCVD. Were they previously diagnosed by ophthalmologist?
5. Table 2: We know that during this age, boys undergo various hormonal changes which could influence their behaviour and emotions. Could you clarify whether the scores might have been influenced by the hormonal changes associated with puberty?
6. Line 168:Among adolescent boys with CCVD, there were statistically significant relationships between their parental education level and behavioural outcomes on both narrow- and broad-band CBCL scales; is there any supplementary table for such analysis?
7. Line 190: You reported similar findings regarding parents’ household income and problems identified in the narrow-band scale. Since association with socio-economic factors was one of your objectives, Formulation and demonstration of this table is important for better clarification.
8. As the scores for Internalizing, Externalizing, and Total Problems are interdependent and derived from the narrow-band scales, the results need to be adjusted using an appropriate statistical test to determine the actual significance. Otherwise, a different statistical analysis should be performed.
9. Did parents who were previously aware of their child’s vision problems seek professional eye care services? Did prior awareness have any impact on their scores?

**Discussion**

1. Has this type of study been conducted only in Malaysia, or also elsewhere in the world? You may consider including such studies in your references to compare your results.
2. I think you could mention in limitation that, since the reporting was done by parents, it may be subject to bias. All the identified adolescents should ideally undergo a mental health screening program.

**Conclusion:**

Do you have any recommendations to include at the end of your conclusion?
