# Supplementary material for: Behavioural and emotional assessment of Malaysian adolescent boys with congenital colour vision deficiency and its association with socioeconomic factors
Source: PLoS One. 2026 Jul 23;21(7):e0353847. doi: 10.1371/journal.pone.0353847 (PMC13395452; doi:10.1371/journal.pone.0353847)
Supplement: Attachment — Submitted filename: REVIEW-Behavioural-091025.docx [file pone.0353847.s002.docx]

**Behavioural and Emotional Issues in Malaysian Adolescent Boys with Congenital Colour Vision Deficiency and Their Association with Socioeconomic Factors.**

PLOS ONE:

1. Abstract and Introduction

Strengths

- The abstract clearly summarizes the research question, methods (cross-sectional comparison), sample size, statistical tests, and key findings (higher behavioural and emotional issues among boys with CCVD and the role of socioeconomic factors).
- The introduction contextualizes the study well by referencing global prevalence data (lines 3–5) and literature on psychosocial impacts of CCVD in children.
- Prior research by the same group (Thomas et al., 2018) is appropriately cited and linked to justify the new focus on adolescents.

Weaknesses

- The introduction, while comprehensive, could better emphasize novelty—how this study adds to prior work beyond replication with an older cohort.
- Some minor redundancy exists when describing the academic and social impact of CCVD (lines 6–20, 21–31). Streamlining could improve clarity.

Verdict: Suitable for publication with minor editorial polish.

2. Figures and Tables

Evaluation

- Tables 1–4 (pages 15–18) are clear, labeled correctly, and include essential statistical information.
- Mean and standard deviations are reported alongside Mann–Whitney U and chi-square results—consistent with best practices.
- Axes are not relevant (no figures/graphs presented), but the table captions are complete and self-explanatory.
- The presentation aligns with the type of data (psychometric T-scores, categorical analysis).

Verdict: Figures/tables meet journal standards. No revision needed.

3. Methods

Strengths

- The methodology is technically sound and well-detailed:
  - Ethical approvals from relevant bodies (page 13, lines 130–135) are listed.
  - The use of Ishihara and Farnsworth D15 tests for CCVD confirmation ensures diagnostic accuracy.
  - CBCL/4–18 Malay version is validated and described (lines 98–112).
  - Clear inclusion/exclusion criteria and parental consent procedures.

Weaknesses

- While statistical methods (Mann–Whitney U, Chi-square, Cramer’s V) are appropriate, power analysis details (effect size assumption, α level) are not explicitly stated.
- Although socioeconomic variables were considered, potential confounders such as parental mental health or school type were not discussed.

Verdict: Methodology is robust and reproducible but needs minor clarifications on sample power justification and confounding control.

4. Results, Discussion, and Conclusions

Results

- Data presentation is clear and logically structured.
- Results support the conclusions without overreach.
- Effect sizes (φ, Cramer’s V) are correctly reported—strengthening statistical transparency.

Discussion

- Thoroughly integrates findings with existing literature (pages 19–22), identifying consistency with previous child studies.
- Thoughtfully explains age-related progression of psychosocial impacts.
- Highlights socioeconomic moderation convincingly with relevant local (Malaysian) citations.
- Limitations are honestly acknowledged—gender restriction, parent-only reports, and test sensitivity.

Conclusion

- Concise, evidence-based, and directly answers the research question.
- Appropriately suggests policy and educational implications (e.g., school screening).

Verdict: Results and discussion are comprehensive and publication-ready.

5. Statistical Analysis

- Statistical tests match data characteristics (nonparametric, small sample).
- Effect sizes are provided, enhancing interpretability.
- However, the absence of confidence intervals and lack of correction for multiple comparisons could be mentioned as limitations.

Verdict: Adequate; minor statistical transparency improvements recommended.

6. Data and Supporting Information

- Data availability is compliant with PLOS ONE policy, with dataset link provided via Figshare.
- Ethical clearance and consent procedures are documented.

Verdict: Fully meets open-data and ethical requirements.

7. Writing Quality and References

- Writing is fluent and scientifically clear.
- Minor grammatical redundancies (e.g., repeated “adolescent boys with CCVD”) could be condensed.
- Reference list is comprehensive, current (includes 2023–2024 sources), and formatted correctly.

Verdict: Clear and scholarly writing; only minor language editing suggested.

| Review Criterion | Assessment | Notes |
| --- | --- | --- |
| Abstract and Intro | Acceptable | Minor stylistic refinements |
| Methods | Sound | Clarify power and confounders |
| Results and Discussion | Strong | Excellent structure and linkage |
| Figures/Tables | Clear | Well-prepared |
| Statistical Analysis | Adequate | Add CI/multiple-test caution |
| Data and Ethics | Complete | Meets journal policy |
| Writing and References | Clear | Minor language edits |

### **Final Recommendation: MINOR REVISION**

The revised manuscript successfully addresses the earlier review concerns. The study is **methodologically sound, well-contextualized, and ethically compliant**. Only **minor revisions**, mainly stylistic and explanatory are required before acceptance.
