# Supplementary material for: Behavioural and emotional assessment of Malaysian adolescent boys with congenital colour vision deficiency and its association with socioeconomic factors
Source: PLoS One. 2026 Jul 23;21(7):e0353847. doi: 10.1371/journal.pone.0353847 (PMC13395452; doi:10.1371/journal.pone.0353847)
Supplement: Attachment — Submitted filename: Response to Reviewers.docx [file pone.0353847.s006.docx]

**Behavioural and Emotional Issues in Malaysian Adolescent Boys with Congenital Colour Vision Deficiency and Their Association with Socioeconomic Factors.**

**Response to Reviewers:**

We sincerely thank the Reviewers for their careful evaluation of our manuscript. All reviewer comments have been addressed. Changes made to the manuscript are indicated with page and line numbers in the revised version. The manuscript has been revised for clarity, transparency, and completeness without altering the core findings or conclusions. We believe these revisions have strengthened the manuscript and thank the reviewers for their valuable input. Both reviewers have provided their comments within the email from the Editor and in separate Word files. As such, we have structured our replies and feedback accordingly.

**Comments on Word File**

| **Reviewer’s Response** | | | | **Author’s Reply** |
| --- | --- | --- | --- | --- |
| **Section** | **Comment** | | |  |
| **Title** | The authors used a behavioral checklist, so the title should reflect the behavioral assessment of Malaysian adolescent boys with congenital color vision deficiency and its association with socio-demographic factors. | | | The title has been revised to reflect the behavioural assessment approach used in this study while maintaining focus on the study population and outcomes.  Current title:  Behavioural and Emotional Issues in Malaysian Adolescent Boys with Congenital Colour Vision Deficiency and Their Association with Socioeconomic Factors  Revised title:  *“Behavioural and Emotional Assessment of Malaysian Adolescent Boys with Congenital Colour Vision Deficiency and Its Association with Socioeconomic Factors.”* |
| **Abstract** | 1. You have not assessed academic performance in your study. Therefore, it may be misleading to state at the beginning that ‘Congenital colour vision deficiency (CCVD) can impact academic performance and social interactions”. | | | As academic performance was not directly assessed in the present study, the wording in the Abstract has been revised to remove reference to academic performance and to better align with the outcomes measured.  Revised abstract opening sentence (REPLACED ORIGINAL):  *“Congenital colour vision deficiency (CCVD) is associated with challenges in daily functioning and social interactions, yet its behavioural and emotional implications during adolescence remain underexplored.”* |
|  | 2. Why was this not conducted as a case-control study? Please justify your choice of study design. | | | This study was not designed as a case–control study because participants were selected based on exposure status (CCVD vs. normal colour vision) rather than on the presence or absence of behavioural/emotional problems. In this study, CCVD is an existing characteristic and is not a past exposure. What was presently assessed was the participants’ behaviour through parental report. Thus, it was not a case-control study.  We have revised first sentence in Materials and Methods to make our study design clearer (page 4, line 40):  *“This prospective, cross-sectional school-based study employed purposive sampling* ….” |
| **Introduction** | Line 32: You have not mentioned any comment regarding socio-economic factors in the objectives section of the introduction. “Thus, the present study aimed to assess the behavioural and emotional issues and challenges faced by secondary school children with CCVD based on parental reports. These findings were compared to those of age-matched peers with NCV across ten secondary schools in Kuala Lumpur, Malaysia.” | | | The Introduction has been revised to explicitly include socioeconomic factors as part of the study objectives.  Added paragraph at end of Introduction (page 4, line 34):  *“Furthermore, this study explored the role of socioeconomic factors in moderating behavioural outcomes among adolescents with CCVD within a Malaysian urban school context.”* |
| **Materials and methods** | | **Study design and participants** | Why was this not conducted as a case-control study? Please justify your choice of study design. | As mentioned in our earlier reply, this study was not designed as a case–control study because participants were selected based on exposure status (CCVD vs. normal colour vision) rather than on the presence or absence of behavioural/emotional problems. In this study, CCVD is an existing characteristic and is not a past exposure. What was presently assessed was the participants’ behaviour through parental report. Thus, it was not a case-control study.  Revised first sentence in Materials and Methods (page 4, line 40):  *“This prospective, cross-sectional school-based study employed purposive sampling ….”* |
|  |  | **Behavioral and emotional assessment:** | Line 112: it is mentioned that “Parents of the identified adolescents completed the CBCL questionnaire and returned it within one week of distribution”. Did you distribute all the forms in a parents’ assembly on the same day or were they distributed individually? Did you demonstrate to them how to fill out the form? Please describe this process clearly in the methodology section. | The methodology section has been expanded to clearly describe the questionnaire distribution and instruction process.  Added the following paragraph under Behavioural and emotional assessment (page 7, lines 123-127):  *“The CBCL were distributed individually to parents of the identified adolescents through the participating schools. Parents were provided with written instructions explaining how to complete the questionnaire and were encouraged to contact the research team if clarification was required. No group demonstration sessions were conducted to minimise response bias. Completed questionnaires were returned to the school within one week of distribution.”* |
|  |  | **Statistical analysis:** | Please mention whether any adjustment was made to determine the significant p-value from the interrelated scores. | To account for multiple comparisons arising from the interrelated CBCL scales and syndromes, we applied the Benjamini–Hochberg False Discovery Rate (FDR) correction. FDR-adjusted q-values have now been added to the tables, and the statistical analysis section in Methods section has been updated accordingly.  We have added this sentences at the end of the last paragraph in Statistical analysis section in Methods (Page 7, lines 143-146):  *“Given the interrelated nature of the CBCL/4-18 scales and syndromes, adjustment for multiple comparisons was performed using the Benjamini–Hochberg False Discovery Rate (FDR) method. Statistical significance was determined based on FDR-adjusted q-values.”*  We have also added these sentences to Results (page 10, lines 176-178):  *“Normality testing with the Shapiro-Wilk test indicated that all mean scores were not normally distributed (p<0.05 for all scales). Therefore, the Mann-Whitney U test was applied for group comparisons. Statistical significance was determined using FDR-adjusted q-values, with q < 0.05 considered statistically significant, as described in the Methods.”*  We have also added FDR adjusted q-value (labelled as q-value) columns to Tables 3,4, 5,6,7 |
| **Result:** | 1. Line 137: “Of the 2,879 adolescent boys who underwent vision screening, 44 (1.53%) were identified as having CCVD, and all of them were deutans.” Which classes were they in, and how old were they? All of them were between 11 and 13 years of age? Is If yes, was there any specific reason for choosing the age group 11–13 years?Please provide a suitable explanation. | | | In Malaysia, secondary schooling starts when children turn 13. Thus, the age range of the adolescents screened was between 13 and 16. The corresponding age-based classes (called ‘Form’ in Malaysia) were 1, 2, 3, and 4, respectively. The age range and school level have now been clarified in the Results and Methods sections.  We have added the following sentence to Results (page 9, lines 155-157:  “*All identified adolescents with CCVD were aged between 13 to 16 years and were enrolled in secondary school (Forms 1, 2, 3 and 4), reflecting the age range targeted during the school-based screening programme.”*  We have also added justification to Methods under the *Recruitment and Consent* section (page 5, lines 58-63):  “*The 13–16-year age group was selected to enable the assessment of behavioural and emotional difficulties during the secondary school years; a developmental period associated with increasing academic and social demands. Moreover, adolescents in this age range are likely cognitively mature enough to comprehend their colour vision status, which may support informed educational and future career decisions.”* |
|  | 2. You mentioned that 40 samples are needed in each group to maintain adequate statistical power. In the results section, you reported 44 boys with congenital colour vision defect. Was this coincidental, or did you stop screening once you reached the desired sample size? Please provide a suitable explanation in the methodology or results section. | | | The required sample size was calculated a priori, with an additional 10% allowance for potential non-response or dropout. Screening was conducted among all eligible students within the selected schools, and recruitment was not terminated upon reaching the minimum required sample size.  We have added this final sentence to the last paragraph in section Study design and participants (page 5, lines 54-55):  *“Screening was conducted on all eligible students in the selected schools, and recruitment was not terminated upon reaching the minimum required sample size.”* |
|  | 3. No need to mention gender of mother in the table 1. | | | We agree with the reviewer.  Action taken:  We have removed the gender of the mother from Table 1. |
|  | 4. Line 143: Only 12 out of 44 mothers of boys with CCVD (27.3%) were aware of their son’s CCVD. Were they previously diagnosed by ophthalmologist? | | | The manuscript has been clarified to indicate that parental awareness of their child’s colour vision difficulties, not CCVD. In the questionnaire given to the parents, they were asked whether they were aware that their children had difficulties with colour-based tasks. Thus, awareness was inferred from parental observations rather than a formal diagnosis of CCVD.  We have added this sentence in Results (page 9, lines 162-165:  *“Only 12 out of 44 mothers of boys with CCVD (27.3%) were aware of their son’s colour vision difficulties, based on parental observations of colour-related difficulties and/or a known family history of colour vision deficiency, rather than prior formal diagnosis by an eye care professional.”* |
|  | 5. Table 2: We know that during this age, boys undergo various hormonal changes which could influence their behaviour and emotions. Could you clarify whether the scores might have been influenced by the hormonal changes associated with puberty? | | | We agree that behavioural and emotional functioning during late childhood and adolescence may indeed be influenced by age-related developmental and pubertal changes. To minimise this potential confounding effect, children with CCVD were compared with closely age-matched children with normal colour vision. As a result, developmental influences common to both groups would be expected to affect CCVD and NCV children similarly.  Consequently, while age-related developmental factors may have influenced behavioural scores overall, they are unlikely to account for the observed group differences. The higher CBCL/4–18 scores observed in children with CCVD may therefore reflect additional influences associated with CCVD that are not present in age-matched children with normal colour vision.  We acknowledge that pubertal stage was not directly assessed, and residual confounding cannot be entirely excluded; this has been noted as a limitation in the revised manuscript.  In Discussion (page 18, lines 262-270):  *“Behavioural and emotional functioning during adolescence may be influenced by developmental and pubertal changes [35,36]. However, in the present study, adolescent boys with CCVD were compared with age-matched peers with normal colour vision, thereby minimising the potential confounding effects of pubertal development. Developmental influences common to this age group would therefore be expected to affect both groups similarly. Accordingly, while age-related factors may have influenced CBCL scores overall, they are unlikely to fully explain the higher behavioural and emotional problem scores observed in the CCVD group. These findings suggest that factors associated with CCVD may contribute to the elevated behavioural and emotional difficulties identified.”*  We have also added to Limitation (page 21, lines 354-358):  *“Second, pubertal status and hormonal influences, which may affect behavioural and emotional regulation during adolescence, were not directly assessed. Although age-matching was used to minimise developmental confounding, residual effects of pubertal variability cannot be entirely excluded and may have contributed to variability in CBCL/4-18 scores.”* |
|  | 6. Line 168:Among adolescent boys with CCVD, there were statistically significant relationships between their parental education level and behavioural outcomes on both narrow- and broad-band CBCL scales; is there any supplementary table for such analysis? | | | A table summarizing the association between parental education level and CBCL outcomes among adolescents with CCVD has been added.  Added *“Table 5: Associations between adolescent boys with CCVD’s parental education level and T-score category for each CBCL/4-18 scale”* |
|  | 7. Line 190: You reported similar findings regarding parents’ household income and problems identified in the narrow-band scale. Since association with socio-economic factors was one of your objectives, Formulation and demonstration of this table is important for better clarification. | | | A table summarizing the association between parents’ household income and behavioural outcomes.  Added *“Table 6: Associations between adolescent boys with CCVD’s parental monthly household income and T-score category for each CBCL/4-18 scale”* |
|  | 8. As the scores for Internalizing, Externalizing, and Total Problems are interdependent and derived from the narrow-band scales, the results need to be adjusted using an appropriate statistical test to determine the actual significance. Otherwise, a different statistical analysis should be performed. | | | To account for multiple comparisons arising from the interrelated CBCL scales and syndromes, we applied the Benjamini–Hochberg False Discovery Rate (FDR) correction. FDR-adjusted q-values have now been added to the tables, and the statistical analysis section in Methods section has been updated accordingly.  We have added this sentences at the end of the last paragraph in Statistical analysis section in Methods (Page 7, lines 143-146):  *“Given the interrelated nature of the CBCL/4-18 scales and syndromes, adjustment for multiple comparisons was performed using the Benjamini–Hochberg False Discovery Rate (FDR) method. Statistical significance was determined based on FDR-adjusted q-values.”*  Added to Results:  *“Normality testing with the Shapiro-Wilk test indicated that all mean scores were not normally distributed (p<0.05 for all scales). Therefore, the Mann-Whitney U test was applied for group comparisons. Statistical significance was determined using FDR-adjusted q-values, with q < 0.05 considered statistically significant, as described in the Methods.”*  Action taken:  *Added FDR adjusted q-values (as q-value columns) to Tables 3,4, 5,6,7.* |
|  | 9. Did parents who were previously aware of their child’s vision problems seek professional eye care services? | | | No, parental awareness of their child’s colour vision deficiency was not based on formal clinical diagnosis. Instead, awareness was inferred from parental observations of the child’s difficulties with colour-related tasks and/or a known family history of colour vision deficiency.  The manuscript has been clarified to indicate this.  Added this sentence immediately after this sentence in Results:  *“Only 12 out of 44 mothers of boys with CCVD (27.3%) were aware of their son’s colour related difficulties, based on parental observations of colour-related difficulties and/or a known family history of colour vision deficiency, rather than prior formal diagnosis by an eye care professional.”* |
|  | Did prior awareness have any impact on their scores? | | | The manuscript has been clarified to include association between parental awareness of their child’s CCVD status and CBCL outcomes among adolescents with CCVD has been added.  Action taken:  Added *“Table 7: Associations between adolescent boys with CCVD’s parental awareness of their child’s CCVD status and T-score category for each CBCL/4-18 scale”*  We have added this paragraph to Results section (page 16, lines 235-246):  *“Within the CCVD group, parental awareness of their child’s CCVD status (unaware vs. aware) was significantly associated with selected behavioural problems on the CBCL/ 4-18 narrow-band scales (Table 7). Adolescents whose parents were unaware of their CCVD status were more likely to score within the borderline clinical/clinical range for Withdrawn (χ²(1) = 6.919, φ = 0.397, p < 0.05, 95% CI = [0.000, 0.054], Thought Problems (χ²(1) = 6.919, φ = 0.397, p < 0.05, 95% CI = [0.000, 0.054]), and Attention Problem (χ²(1) = 7.700, φ = 0.418, p < 0.05, 95% CI = [0.000, 0.054]). Notably, all parents who were aware of their child’s CCVD status (n = 12) rated their adolescents within the normal range across all CBCL/4–18 narrow-band scales, whereas elevated scores were observed exclusively among adolescents whose parents were unaware of the condition. No statistically significant associations were observed between parental awareness and broad-band scales (Internalizing, Externalizing, and Total Problems) (p > 0.05).”*  We have also discussed these findings in Discussion section (page 18, lines 271-280):  *“An important finding of this study was that elevated behavioural scores were observed exclusively among adolescents whose parents were unaware of their CCVD status, whereas all adolescents with parental awareness were rated within the normal range across narrow-band scales. This suggests that parental awareness, despite the absence of formal clinical diagnosis, may influence how behavioural problems are perceived and interpreted. Recognition of CCVD-related functional limitations may reduce misattribution of associated difficulties to behavioural or emotional problems. Alternatively, differences in behavioural ratings may reflect variation in parental perception rather than true differences in symptom severity.”*  We have also added this sentence in Limitations (page 22, lines 361-365):  *“Fourth, parental awareness was not based on prior clinical diagnosis but on observation or family history, which may have affected behavioural ratings. Additionally, the small number of parents reporting awareness limited subgroup analyses. Future studies incorporating clinically confirmed diagnoses and multi-informant assessments would strengthen interpretation of behavioural outcomes.”* |
| **Discussion** | 1. Has this type of study been conducted only in Malaysia, or also elsewhere in the world? You may consider including such studies in your references to compare your results. | | | We have revised our Discussion section to include studies in reference to compare our result (page 19, lines 289-301).  *In line with these findings, literature highlights the broader behavioural and emotional impact of CCVD across childhood and adolescence. Although children with CCVD often appear developmentally typical, repeated difficulties with colour-dependent academic tasks can lead to frustration, embarrassment, and diminished self-confidence when these challenges are misunderstood by adults or peers [4,33,34]. Beyond academics, difficulties in sports and play, including distinguishing team colours or tracking objects against similarly coloured backgrounds, may further impair peer interactions and social participation [4]. Over time, such repeated experiences of failure and misunderstanding may contribute to internalising behaviours, withdrawal, and externalising responses, particularly during adolescence when academic demands and social expectations intensify. Reviews and commentaries further suggest that delayed identification of CCVD may negatively affect well-being, self-esteem, and future educational or career choices, with late diagnosis sometimes triggering psychological distress, anger, or grief [6,20].* |
|  | 2. I think you could mention in limitation that, since the reporting was done by parents, it may be subject to bias. All the identified adolescents should ideally undergo a mental health screening program. | | | We agree and have expanded the Limitations section accordingly.  Added to Limitations:  *“Incorporating adolescent self-reports or teacher evaluations along with structured mental health screening, in future studies would enhance the findings.”* |
| **Conclusion:** | Do you have any recommendations to include at the end of your conclusion? | | | Practical recommendations have been added to the Conclusion.  Added to Conclusion (page 23, lines 381-383):  *“Routine school-based colour vision screening, coupled with early behavioural and emotional assessment and targeted educational support, may help mitigate long-term psychosocial challenges among adolescents with CCVD.”* |

**Comments on Email**

| **Review Comments to the Author: Reviewer #1** | **Author’s Response** |
| --- | --- |
| Thank you for your research initiative on adolescent boys with congenital colour vision deficiency (CCVD). I have a few suggestions to further strengthen your findings. | We thank Reviewer #1 for the detailed, balanced, and supportive evaluation of our work. |
| Has similar work been conducted previously on CCVD? Please consider adding a few more references. | Has been address above. |
| I also believe there is an opportunity to review and potentially revise the title. | We appreciate this suggestion. After careful consideration, we have slightly refined the title to improve clarity while maintaining consistency with the study scope.  Revised title:  “*Behavioural and Emotional Assessment of Malaysian Adolescent Boys with Congenital Colour Vision Deficiency and Its Association with Socioeconomic Factors.”* |
| A major table demonstrating CBCL/4-18 T scores alongside socioeconomic factors was not included in your file. Please upload your data as a supplementary file for better clarification. | A table presenting the association between parental education level and monthly household income with CBCL outcomes among adolescents with CCVD has been added.  Action taken:  Added *“Table 5: Associations between adolescent boys with CCVD’s parental education level and T-score category for each CBCL/4-18 scale”*  Added *“Table 6: Associations between adolescent boys with CCVD’s parental monthly household income and T-score category for each CBCL/4-18 scale”* |
| I have a query regarding the study design: why was this not conducted as a case-control study?  Since the scores for Internalizing, Externalizing, and Total Problems are interdependent and derived from the narrow-band scales, the results should be adjusted using an appropriate statistical test to determine their actual significance.  Please check the attached file for other modifications. | Has been addressed above.  All editorial and minor textual suggestions provided in the attached file have been carefully reviewed and incorporated into the revised manuscript. |
| **Review Comments to the Author: Reviewer #2** | **Author’s Reply** |
| This is a technically sound cross-sectional case–control study comparing behavioural and emotional problems in adolescent boys with CCVD versus age-/class-matched peers with normal colour vision. Diagnostic confirmation (Ishihara + Farnsworth D15), validated outcomes (CBCL/4–18 Malay), and appropriate nonparametric and categorical statistics make the analysis rigorous. Findings (elevated narrow-band scales including Anxious/Depressed, Withdrawn, Thought Problems, Attention Problems, Delinquent, Aggressive; plus higher Externalizing and Total Problems) are reported transparently and support the conclusions.  Major strengths  1. Clear design and recruitment across 10 schools with matching by age and class; sample size rationale is given. 2. Rigorous phenotyping (Ishihara under controlled luminance and D15 classification with defined error criteria). 3. Validated outcomes & scoring with clinical/borderline thresholds described. 4. Appropriate statistics with effect sizes, and results that align with conclusions (including socioeconomic associations within the CCVD subgroup). 5. Ethics approvals and open data comply with PLOS policy.   Suggestions for minor revision (clarifications/editorial)  Multiple comparisons: You test many CBCL scales. Please add a brief statement acknowledging no formal multiplicity adjustment (or provide justification if treating this as an exploratory multi-endpoint study).  Consider adding confidence intervals for key effect sizes to aid interpretation.    Power justification: You state a target of n=44 per group from prevalence considerations; please add a sentence on the anticipated effect size and α used to deem this sample size adequate.  Reporter/source of outcome: Outcomes rely solely on parent (mother) report. Add a line in Limitations about possible reporting bias and the value of multi-informant data (e.g., youth self-report/teacher ratings) in future work—this is already hinted but could be made explicit.  Phenotyping sensitivity: Note that D15 primarily detects moderate–severe defects; very mild CCVD may be under-detected. You mention criteria—just add a succinct limitation sentence to signal awareness.  Editorial polish:  In the ethics statement, there is a small mixed-language connector (“dan the Department of Education…”)—please correct to “and the Department…”.  Consider trimming small redundancies in the Introduction where impacts of CCVD on schooling are reiterated to improve flow.  Conclusion & recommendation: Your results are consistent, methods are sound, and conclusions are appropriately circumscribed for a cross-sectional study. I recommend Minor Revision to incorporate the clarifications above. | We thank Reviewer #2 for the detailed, balanced, and supportive evaluation of our work.  **`**  Has been address above  We have recalculated and included 95% confidence intervals for key effect size estimates in the Results section to provide additional precision and aid interpretation of the observed associations. This has also been clarified in the Statistical Analysis section of the Methods and reflected in Results (associations analysis).  We have added a sentence in Statistical analysis under Materials and Methods section (page 8, lines 137-138):  *“Effect sizes for the Chi-square analyses were reported using Cramer’s V, along with 95% confidence intervals to provide additional precision in interpretation.”*  We agree with this comment. The sample size of 40 per group (plus 10% to account for dropouts, n=44) was calculated to detect a large effect size (Cohen’s w = 0.6) with 80% power at α = 0.05, which is sufficient for the planned analyses.  Thus, we have added a sentence in Study design and participants under Materials and Methods section (page 5, lines 52-54):  *“This sample size provides 80% power to detect a large effect size (Cohen’s w = 0.6) at a significance level of α = 0.05, which is considered adequate for the planned analyses.”*  We agree and have strengthened the Limitations section to explicitly note reliance on parent (predominantly maternal) reports and the potential value of multi-informant assessments in future studies.  Expanded existing limitation paragraph (page 22, lines 358-361):  *“Third, the behavioural assessment using CBCL/4-18 relied solely on parent reports, which may be subject to reporting bias or under-reporting. Incorporating adolescent self-reports or teacher evaluations together with structured mental health screening, in future studies would enhance the findings.”*  We have added a concise statement acknowledging that very mild CCVD may not be detected by D15 testing and that this may have led to underestimation.  Expanded existing limitation paragraph (page 22, lines 365-367):  *“Fifth, the Farnsworth D15 test used in this study primarily detects moderate to severe CCVD, which may have resulted in under-identification of adolescents with mild colour vision deficiencies.”*  This has been corrected to “and the Department of Education.”  Replaced:  “dan the Department of Education”  With:  “and the Department of Education”  We have streamlined overlapping descriptions of academic and social impacts of CCVD to improve flow and readability.  Revised Introduction section (page 1):  *“Individuals with congenital colour vision deficiency (CCVD) experience difficulties discriminating red and green hues. The condition is more prevalent in males than in females due to its X-linked inheritance pattern. It affects approximately 3-9% of males and 0.1-0.9% of females in most populations [1–3].*  *Although CCVD is a non-progressive condition, its effect on daily life experiences is considerable, which may negatively affect independence and self-esteem. In the classroom, students with CCVD also face additional challenges in tasks requiring colour discrimination, such as reading colour-coded maps, graphs, charts, and using coloured instructional materials [4,5]. Often, children with CCVD may remain unaware of their condition until they start school and face colour-dependent tasks [6].*  *The delay in CCVD diagnosis may impair a child’s ability to cope with CCVD, which would affect their emotional and behavioural development. Primary school children with CCVD are reported to experience more behavioural and emotional problems than their peers with normal colour vision (NCV) [7]. Specifically, these children were reported to display higher levels of aggression, withdrawal, thought-related issues, and delinquent behaviour. Unfortunately, colour vision tests are not always included in routine vision screening tests [8], which can delay CCVD diagnosis and hinder timely intervention.*  *Without timely intervention, these challenges may persist into adolescence, leading to more pronounced social isolation, anxiety, and depressive symptoms [4,9]. Older children with CCVD reported continued difficulties in colour-related educational and daily activities [5]. They may also be perceived as uncooperative and inattentive, resulting in frustration, embarrassment, and ridicule from both peers and teachers [10]. Furthermore, older children with CCVD who remain undiagnosed may face additional challenges when career choices requiring normal colour vision become unattainable [11,12]*  *We previously reported that primary school children with CCVD were perceived to be emotionally sensitive, socially withdrawn, to have a shorter attention span, and to demonstrate poorer academic performance compared to their peers with NCV [7]. However, less is known about the behavioural and emotional impact of CCVD during secondary schooling, which is characterised by increased academic demands and psychosocial challenges. Thus, the present study aimed to assess the behavioural and emotional issues and challenges faced by secondary school children with CCVD based on parental reports. These findings were compared to those of age-matched peers with NCV across ten secondary schools in Kuala Lumpur, Malaysia. Furthermore, this study uniquely explores the role of socioeconomic factors in moderating behavioural outcomes among adolescents with CCVD within a Malaysian urban school context”.* |
