# Supplementary material for: Behavioural and emotional assessment of Malaysian adolescent boys with congenital colour vision deficiency and its association with socioeconomic factors
Source: PLoS One. 2026 Jul 23;21(7):e0353847. doi: 10.1371/journal.pone.0353847 (PMC13395452; doi:10.1371/journal.pone.0353847)
Supplement: Attachment — Submitted filename: CCVD.PLOS.docx [file pone.0353847.s004.docx]

The authors have addressed the necessary advice and corrections. However, a few aspects still require improvement.

1. **Lines 161–165:** Please rewrite this section to avoid duplication.
2. **Below Table 3:** Please include the significant **q values** in addition to the **p values**.
