# Supplementary material for: Behavioural and emotional assessment of Malaysian adolescent boys with congenital colour vision deficiency and its association with socioeconomic factors
Source: PLoS One. 2026 Jul 23;21(7):e0353847. doi: 10.1371/journal.pone.0353847 (PMC13395452; doi:10.1371/journal.pone.0353847)
Supplement: Attachment — Submitted filename: Recommendation.docx [file pone.0353847.s005.docx]

Recommendation – 2^nd^ Review

Major Revision

Q2. Is the manuscript technically sound, and do the data support the conclusions?

Partly. The study question is clear and the overall approach (screening-confirmed CCVD, matched comparison group, CBCL outcomes, and subgroup socioeconomic analyses) is reasonable. However, the current submission contains substantial presentation problems (tracked-changes artefacts) and several internal inconsistencies that need correction before the technical merits can be fully assessed (see detailed comments). For example, the Abstract and main text display “Formatted: Highlight / Revised Manuscript with Track Changes” artefacts, which is not acceptable for peer review or publication.

Q3. Has the statistical analysis been performed appropriately and rigorously?

Partly. The use of Shapiro–Wilk for small samples and nonparametric group comparisons is appropriate, and the manuscript states that multiple-comparison adjustment was performed using Benjamini–Hochberg FDR with inference based on q-values. However, reporting is inconsistent and sometimes conflates p-values and q-values (e.g., “all qp<0.05” and “q<0.05p<0.05”), and the effect-size/CI reporting claim in Methods is duplicated and appears not to be implemented consistently across all relevant tables.

Q4. Have the authors made all data underlying the findings fully available?

Yes (as stated), but please verify completeness. The submission includes a Figshare repository link for the underlying data. Before acceptance, please ensure that the repository contains the de-identified, individual-level dataset underlying *all* analyses (e.g., CBCL scale scores/T-scores or raw-to-T transformation inputs as applicable, group indicators, and socioeconomic variables), plus a clear data dictionary/codebook and any analysis syntax needed to reproduce results.

Q5. Is the manuscript presented intelligibly and written in standard English?

No. The file appears to contain tracked-changes/Word formatting artefacts embedded in the PDF (e.g., “Formatted: Highlight / Revised Manuscript with Track Changes”), including in the Abstract. There are also visible typographical issues (e.g., “dan and”; spacing/duplication; stray characters). A clean, publication-ready version (without tracked changes and without Word “Formatted:” tags) is required.

Q6. Review Comments to the Author

Summary

This manuscript addresses an under-studied topic: behavioural and emotional outcomes among adolescents with congenital colour vision deficiency (CCVD) and the role of socioeconomic context. The general design (school screening with diagnostic confirmation, matched controls, and CBCL-based outcomes) is potentially suitable for PLOS ONE. However, the current revision cannot be evaluated properly due to major presentation issues (tracked-changes artefacts) and several internal inconsistencies in reporting and terminology.

Major issues requiring revision

1. Submit a clean manuscript (no tracked changes / no Word formatting artefacts).
   The manuscript includes “Formatted: Highlight” and “Revised Manuscript with Track Changes” text inside the body, including the Abstract. Please upload a clean version with all tracking removed and formatting tags eliminated.
2. Resolve Abstract duplication and ensure content matches measured outcomes.
   The Abstract contains duplicated opening sentences and mixed wording. Please remove duplication and ensure statements align strictly with outcomes assessed in this study.
3. Clarify and standardize multiplicity control and statistical reporting (p vs q).
   The Methods state that FDR adjustment (Benjamini–Hochberg) was applied and that significance is based on q-values. Yet the Abstract and Results use confusing combined notations (e.g., “all qp<0.05” and “q<0.05p<0.05”). Action needed: define clearly (once) which values are used for inference, report p and q in separate columns consistently, and avoid merged expressions.
4. Effect sizes and confidence intervals: ensure consistency with what is claimed.
   The Methods state that chi-square effect sizes (Cramer’s V) were reported with 95% confidence intervals, but the sentence is duplicated/concatenated and it is not evident that CI reporting is applied uniformly across all chi-square analyses.
   Action needed: (a) fix the duplicated sentence, and (b) either provide CIs for all reported effect sizes across the relevant results tables, or revise the Methods to match what is actually reported.
5. Study design terminology is inconsistent and must be corrected.
   In the response material, the authors state the study was not designed as a case–control study because participants were selected by exposure status, yet elsewhere it is described as a “cross-sectional case–control study”

.
Action needed: use a single, technically accurate description throughout (e.g., “matched cross-sectional comparative study” or equivalent) and ensure consistent use across manuscript and response text.

6. Data availability: ensure reproducibility beyond a link.
   A Figshare link is provided, which is a good start. Before acceptance, please confirm the repository includes the *full* underlying dataset and sufficient documentation (codebook, variable definitions, handling of missing data, and any transformations to T-scores) to reproduce all tables and inferential tests.

Minor issues and editorial corrections

- Methods/ethics wording: remove typos and duplicated words (e.g., “dan and”) .
- Results text needs proofreading for merged words/phrases (e.g., “difficultiesCCVD”).
- Limitations section has numbering/duplication issues (e.g., “Second, Third”); please correct structure and remove repeated phrases.
- Power/sample size reporting: the accompanying review notes that power analysis assumptions are not fully specified. If a priori power was used, report assumed effect size, alpha (and whether alpha/q was targeted), and allocation. If not, describe justification and interpret results cautiously.

Conclusion

The topic is relevant and the design could meet PLOS ONE’s technical criteria, but the manuscript requires major revision to (1) provide a clean version without tracked changes artefacts, (2) correct inconsistencies in statistical reporting and design terminology, and (3) ensure effect-size/CI reporting and open-data reproducibility are implemented exactly as described.
