# Supplementary material for: Behavioural and emotional assessment of Malaysian adolescent boys with congenital colour vision deficiency and its association with socioeconomic factors
Source: PLoS One. 2026 Jul 23;21(7):e0353847. doi: 10.1371/journal.pone.0353847 (PMC13395452; doi:10.1371/journal.pone.0353847)
Supplement: Attachment — Submitted filename: Rev.2_Authors Reply_PlosOne_4Feb.docx [file pone.0353847.s008.docx]

**Behavioural and Emotional Issues in Malaysian Adolescent Boys with Congenital Colour Vision Deficiency and Their Association with Socioeconomic Factors.**

**Response to Reviewers’ Recommendation (2^nd^ Review):**

We thank the reviewer for their careful assessment of the manuscript and for recognising that the study question, design, and overall analytical approach are appropriate. All specific inconsistencies highlighted in the detailed comments have now been addressed. In the revised submission, all tracked changes, highlights, and formatting artefacts have been fully removed, and the manuscript has been carefully proofread to ensure consistency across the Abstract, Methods, Results, and Discussion sections. We hope that, with these presentation issues resolved, the study's technical soundness and the alignment between the data and conclusions can now be more clearly evaluated.

**Comments on Word File (attachment)**

| **Reviewer’s Response** | | **Author’s Reply** |
| --- | --- | --- |
| **Section** | **Comment/ Recommendation** |  |
| **Q2. Is the manuscript technically sound, and do the data support the conclusions?** | Partly. The study question is clear and the overall approach (screening-confirmed CCVD, matched comparison group, CBCL outcomes, and subgroup socioeconomic analyses) is reasonable. However, the current submission contains substantial presentation problems (tracked-changes artefacts) and several internal inconsistencies that need correction before the technical merits can be fully assessed (see detailed comments). For example, the Abstract and main text display “Formatted: Highlight / Revised Manuscript with Track Changes” artefacts, which is not acceptable for peer review or publication. | We agree that the previous submission contained presentation-related issues, including residual tracked changes and formatting artefacts, which may have obscured the technical clarity of the manuscript. These issues were unintentional and arose during the revision process.  In the revised submission, all tracked changes, highlights, and formatting artefacts have been fully removed, and the manuscript has been carefully proofread to ensure internal consistency across the Abstract, Methods, Results, and Discussion sections. We have also addressed all specific inconsistencies highlighted in the detailed reviewer comments. |
| **Q3. Has the statistical analysis been performed appropriately and rigorously?** | Partly. The use of Shapiro–Wilk for small samples and nonparametric group comparisons is appropriate, and the manuscript states that multiple-comparison adjustment was performed using Benjamini–Hochberg FDR with inference based on q-values. However, reporting is inconsistent and sometimes conflates p-values and q-values (e.g., “all qp<0.05” and “q<0.05p<0.05”), and the effect-size/CI reporting claim in Methods is duplicated and appears not to be implemented consistently across all relevant tables. | We agree that the description and reporting of p-values and FDR-adjusted q-values required clarification and greater consistency.  In response, we have revised the manuscript to ensure that statistical inference is consistently based on FDR-adjusted q-values, as specified in the Statistical Analysis section (lines 150-153). All instances of ambiguous or conflated reporting have been corrected, and statistical significance has been defined uniformly as q < 0.05.  In addition, we have reviewed and corrected the reporting of effect sizes and confidence intervals to ensure alignment with the Methods description (lines 143-145). Effect sizes are now consistently reported for all chi-square analyses using Cramér’s V, along with 95% confidence intervals for Cramér’s V estimated using nonparametric bootstrapping (1,000 resamples, percentile method). |
| **Q4. Have the authors made all data underlying the findings fully available?** | Yes (as stated), but please verify completeness. The submission includes a Figshare repository link for the underlying data. Before acceptance, please ensure that the repository contains the de-identified, individual-level dataset underlying all analyses (e.g., CBCL scale scores/T-scores or raw-to-T transformation inputs as applicable, group indicators, and socioeconomic variables), plus a clear data dictionary/codebook and any analysis syntax needed to reproduce results. | The dataset in the Figshare repository has been updated. All individuals are anonymous. All data are presented with headings that include the raw score, the T-score, and the socioeconomic variables. There is no missing data. |
| **Q5. Is the manuscript presented intelligibly and written in standard English?** | No. The file appears to contain tracked-changes/Word formatting artefacts embedded in the PDF (e.g., “Formatted: Highlight / Revised Manuscript with Track Changes”), including in the Abstract. There are also visible typographical issues (e.g., “dan and”; spacing/duplication; stray characters). A clean, publication-ready version (without tracked changes and without Word “Formatted:” tags) is required. | This has been addressed above. All tracked changes, highlights, and formatting artefacts have been fully removed. |
| **Q6. Review Comments to the Author – Summary**  **This manuscript addresses an under-studied topic: behavioural and emotional outcomes among adolescents with congenital colour vision deficiency (CCVD) and the role of socioeconomic context. The general design (school screening with diagnostic confirmation, matched controls, and CBCL-based outcomes) is potentially suitable for PLOS ONE. However, the current revision cannot be evaluated properly due to major presentation issues (tracked-changes artefacts) and several internal inconsistencies in reporting and terminology.** | **Major issues requiring revision** | |
|  | 1. Submit a clean manuscript (no tracked changes / no Word formatting artefacts).  The manuscript includes “Formatted: Highlight” and “Revised Manuscript with Track Changes” text inside the body, including the Abstract. Please upload a clean version with all tracking removed and formatting tags eliminated. | This has been addressed above. All tracked changes, highlights, and formatting artefacts have been fully removed. |
|  | 2. Resolve Abstract duplication and ensure content matches measured outcomes.  The Abstract contains duplicated opening sentences and mixed wording. Please remove duplication and ensure statements align strictly with outcomes assessed in this study. | It has been addressed above. All duplications have been removed. |
|  | 3. Clarify and standardize multiplicity control and statistical reporting (p vs q).  The Methods state that FDR adjustment (Benjamini–Hochberg) was applied and that significance is based on q-values. Yet the Abstract and Results use confusing combined notations (e.g., “all qp<0.05” and “q<0.05p<0.05”). Action needed: define clearly (once) which values are used for inference, report p and q in separate columns consistently, and avoid merged expressions. | This has been addressed above. |
|  | 4. Effect sizes and confidence intervals: ensure consistency with what is claimed.  The Methods state that chi-square effect sizes (Cramer’s V) were reported with 95% confidence intervals, but the sentence is duplicated/concatenated, and it is not evident that CI reporting is applied uniformly across all chi-square analyses.  Action needed: (a) fix the duplicated sentence, and (b) either provide CIs for all reported effect sizes across the relevant results tables or revise the Methods to match what is actually reported. | This has been addressed above. The duplicated sentence has been removed. Effect sizes are now consistently reported for all chi-square analyses using Cramér’s V, along with 95% confidence intervals. |
|  | 5. Study design terminology is inconsistent and must be corrected.  In the response material, the authors state the study was not designed as a case–control study because participants were selected by exposure status, yet elsewhere it is described as a “cross-sectional case–control study”.  Action needed: use a single, technically accurate description throughout (e.g., “matched cross-sectional comparative study” or equivalent) and ensure consistent use across manuscript and response text. | We have revised the manuscript to use a single, technically accurate description throughout. The study is now consistently described as a matched cross-sectional comparative study (line 39), as participants were identified through school-based colour vision screening and compared by CCVD exposure status rather than selected based on behavioural outcomes. All references to “case–control” design have been removed from the Abstract, Methods, Results, Discussion, and response materials to ensure consistency and clarity. |
|  | 6. Data availability: ensure reproducibility beyond a link.  A Figshare link is provided, which is a good start. Before acceptance, please confirm the repository includes the full underlying dataset and sufficient documentation (codebook, variable definitions, handling of missing data, and any transformations to T-scores) to reproduce all tables and inferential tests. | This has been addressed above. The dataset in the Figshare repository has been updated. All individuals are anonymous. All data are presented with headings that include the raw score, the T-score, and the socioeconomic variables. There is no missing data. |
|  | **Minor issues and editorial corrections** | |
|  | • Methods/ethics wording: remove typos and duplicated words (e.g., “dan and”) . | This has been addressed above. All types and duplicated words have been rectified. |
|  | • Results text needs proofreading for merged words/phrases (e.g., “difficultiesCCVD”). | This has been addressed above. |
|  | • Limitations section has numbering/duplication issues (e.g., “Second, Third”); please correct structure and remove repeated phrases. | We have corrected the structure and removed repeated phrases in the Limitation section. |
|  | • Power/sample size reporting: the accompanying review notes that power analysis assumptions are not fully specified. If a priori power was used, report assumed effect size, alpha (and whether alpha/q was targeted), and allocation. If not, describe justification and interpret results cautiously. | The sample size was determined a priori based on the expected prevalence of CCVD among male schoolchildren and the feasibility of school-based screening. A minimum of 40 adolescents per group was targeted, assuming a large effect size (Cohen’s w = 0.6), an alpha level of 0.05, and 1:1 group allocation. This sample provides approximately 80% power to detect group differences using Chi-square tests. An additional 10% was added to account for potential non-response or incomplete data, resulting in a final target of 44 participants per group. These can be found in the revised manuscript (lines 52-57).  We also note that adjustment for multiple comparisons using the Benjamini–Hochberg FDR procedure was applied during analysis and was not incorporated into the a priori sample size estimation. Accordingly, results should be interpreted cautiously as exploratory and hypothesis-generating. |
| **The authors have addressed the necessary advice and corrections. However, a few aspects still require improvement.** | 1. Lines 161–165: Please rewrite this section to avoid duplication. | Lines 161–165 have been rewritten and consolidated into a single sentence to improve clarity and avoid repetition.  **Replaced at Line 163-165:**  All identified adolescents with CCVD and age-matched peers with normal colour vision were aged 13–16 years (mean age = 14.52 ± 1.30 years), enrolled in Forms 1–4 of secondary school, and were of Malay ethnicity. |
|  | 2. Below Table 3: Please include the significant q values in addition to the p values**.** | As suggested, the footnote to Table 3 has been revised to clarify that statistical significance was determined using q < 0.05.  **Replaced Line 215-216:**  *Both unadjusted p-values and FDR-adjusted q-values are reported; statistical significance was defined as q < 0.05. |
| **Conclusion**  **The topic is relevant, and the design could meet PLOS ONE’s technical criteria, but the manuscript requires major revision to (1) provide a clean version without tracked changes artefacts, (2) correct inconsistencies in statistical reporting and design terminology, and (3) ensure effect-size/CI reporting and open-data reproducibility are implemented exactly as described.** | | |
