# Supplementary material for: Behavioural and emotional assessment of Malaysian adolescent boys with congenital colour vision deficiency and its association with socioeconomic factors
Source: PLoS One. 2026 Jul 23;21(7):e0353847. doi: 10.1371/journal.pone.0353847 (PMC13395452; doi:10.1371/journal.pone.0353847)
Supplement: Attachment — Submitted filename: Reviewed-190226.docx [file pone.0353847.s007.docx]

My responses to the review questions are based on the points below.

1. Technical soundness and support for conclusions. The study design and measurement tools are generally suitable, and the primary comparisons between CCVD and NCV groups appear consistent with the results presented. However, several reporting and presentation issues reduce confidence in the accuracy of the statistical reporting and prevent the manuscript from being considered publication ready at this stage.
2. Statistical analysis. The choice of non-parametric testing for group comparisons after assessing distributional assumptions is reasonable. The stated plan to control multiplicity using Benjamini to Hochberg false discovery rate is also appropriate, and the tables include both p values and q values. That said, there are inconsistencies that require correction before the reader can interpret results reliably. In particular, the manuscript reports Shapiro Wilk normality findings using q values. Normality tests produce p values, and it is not clear why FDR adjustment would be applied to normality testing. Please correct this to p values, or clearly justify and document any adjustment if it was applied. In addition, subgroup chi square analyses described as within the CCVD group repeatedly report N equal to 88 even though the CCVD group size is 44. Please correct all subgroup chi square statements to the correct N for the subgroup being analysed, and confirm that the analyses were run on the intended subset.
3. Data availability. A Figshare DOI is provided, which is positive and aligns with the journal’s data policy. Before acceptance, please ensure the repository includes the underlying individual level dataset required to reproduce all tables, plus a clear codebook describing variables, coding, and scoring, and sufficient information to reproduce the analyses, including how CBCL scores were handled and transformed where applicable.
4. Presentation and language. The main barrier to intelligibility is the manuscript file itself. The PDF includes duplicated content and material that appears to be tracked changes embedded within the text. This makes the document difficult to read and evaluate as a clean revised version. Please upload a single clean manuscript file with all track change artefacts removed.

Additional suggestions to strengthen clarity and interpretation.
a. Maintain consistent language throughout the Abstract, Results narrative, and tables regarding which values are used for inference. If you state that conclusions are based on q values, ensure the narrative highlights adjusted results consistently and avoids mixing p based and q based interpretations.
b. Because the CCVD subgroup analyses involve small cell counts and multiple comparisons, please frame these findings explicitly as exploratory, and consider reporting effect sizes or risk differences alongside significance testing to support practical interpretation.

Ethics and publication ethics. Ethical approvals are stated. I did not identify clear concerns about research ethics from the information provided. If any part of the dataset cannot be shared publicly due to privacy restrictions, this should be stated explicitly in the Data Availability section, along with the mechanism for controlled access.

In summary, the study appears potentially publishable on technical grounds, but I recommend further revision focused on correcting statistical reporting inconsistencies, ensuring accurate subgroup sample size reporting, providing a clean readable manuscript file, and strengthening reproducibility documentation in the data repository.
