# Supplementary material for: Behavioural and emotional assessment of Malaysian adolescent boys with congenital colour vision deficiency and its association with socioeconomic factors
Source: PLoS One. 2026 Jul 23;21(7):e0353847. doi: 10.1371/journal.pone.0353847 (PMC13395452; doi:10.1371/journal.pone.0353847)
Supplement: Attachment — Submitted filename: Rev.3_Authors Reply_PlosOne_April_v2.docx [file pone.0353847.s010.docx]

**Behavioural and Emotional Issues in Malaysian Adolescent Boys with Congenital Colour Vision Deficiency and Their Association with Socioeconomic Factors**

**Response to Reviewers’ Comments (3^rd^ Review):**

We thank the reviewer for recognising the suitability of the study design and measurement tools. In response to the reviewer’s comments, we carefully reviewed the manuscript to correct reporting inconsistencies and improve clarity. All statistical statements were rechecked against the SPSS output to ensure accuracy and consistency throughout the Abstract, Methods, Results, and tables.

**Comments on Word File (attachment)**

| **Reviewer #2’s comment** | **Authors’ response** | **Amendment in Manuscript** |
| --- | --- | --- |
| **1. Statistical analysis.** | | |
| *1a. The choice of non parametric testing for group comparisons after assessing distributional assumptions is reasonable. The stated plan to control multiplicity using Benjamini to Hochberg false discovery rate is also appropriate, and the tables include both p values and q values. That said, there are inconsistencies that require correction before the reader can interpret results reliably. In particular, the manuscript reports Shapiro Wilk normality findings using q values. Normality tests produce p values, and it is not clear why FDR adjustment would be applied to normality testing. Please correct this to p values, or clearly justify and document any adjustment if it was applied.* | We thank the reviewer for identifying this issue. The Shapiro–Wilk test was used solely to assess distributional assumptions prior to selecting appropriate statistical tests. As correctly noted, normality testing produces p-values and was not subject to false discovery rate (FDR) adjustment. Thus, all Shapiro–Wilk results have been corrected to report p-values only. | The Statistical Analysis section has been revised to state that Shapiro–Wilk tests are interpreted using p-values, while FDR adjustment was applied only to CBCL outcome comparisons (see lines 179-180). |
| *1b. In addition, subgroup chi square analyses described as within the CCVD group repeatedly report N equal to 88 even though the CCVD group size is 44. Please correct all subgroup chi square statements to the correct N for the subgroup being analysed, and confirm that the analyses were run on the intended subset.* | We appreciate the reviewer identifying this issue. The subgroup analyses examining associations between behavioural outcomes and socio-demographic factors were conducted only within the CCVD group (n = 44). Result statements previously displaying N = 88 have been corrected. | All subgroup chi-square result statements have been corrected to N = 44 in the Results section for the: Associations between adolescent boys with CCVD’s parental monthly household income (lines 226-232), parental education level (lines 214-220) and parental awareness of their child’s CCVD status (lines 240-246). |
| *2. Data availability. A Figshare DOI is provided, which is positive and aligns with the journal’s data policy. Before acceptance, please ensure the repository includes the underlying individual level dataset required to reproduce all tables, plus a clear codebook describing variables, coding, and scoring, and sufficient information to reproduce the analyses, including how CBCL scores were handled and transformed where applicable.* | We thank the reviewer for this helpful suggestion. The Figshare repository now includes the de-identified individual-level dataset used to generate all tables in the manuscript. A separate, detailed codebook and documentation describing CBCL score categorisation have also been uploaded to figshare. | The Data Availability statement was updated to confirm that the Figshare repository includes the dataset, codebook, and CBCL scoring documentation.  Regarding the CBCL/4–18 scoring procedure, we have included the statement below in the codebook uploaded to Figshare:  The CBCL is completed by the parent or primary caregiver, who rates each item based on the child’s behaviour over the preceding six months using a 3-point Likert scale (0 = not true, 1 = somewhat or sometimes true, 2 = very true or often true).  Raw scores for each scale are calculated by summing item responses and subsequently converted into T-scores using age- and gender-specific normative tables provided in the ASEBA manual. This conversion is performed manually using standard lookup tables. CBCL T-scores are standardised with a mean of 50 and standard deviation of 10, with separate norms for gender and age groups (4–11 and 12–18 years).  T-scores are then categorised into normal, borderline clinical, and clinical ranges based on established CBCL thresholds (broad-band scales: normal <60, borderline 60–63, clinical >63; narrow-band scales: normal <67, borderline 67–70, clinical >70), as recommended in the ASEBA manual. |
| *3. Presentation and language. The main barrier to intelligibility is the manuscript file itself. The PDF includes duplicated content and material that appears to be tracked changes embedded within the text. This makes the document difficult to read and evaluate as a clean revised version. Please upload a single clean manuscript file with all track change artefacts removed.* | As per PLOS One’s requirements, for the previous submission of the manuscript, we have uploaded both the clean, revised version and the version with tracked changes to the submission system. The PDF that the PLOS One system produced thus contains the clean version followed by the tracked changes version.  We agree that the tracked changes version contains a lot of duplicated text, indicating the changes we made following the prior rounds of revision.  We have now uploaded the tracked changes version without the duplicated text, making it easier for Reviewer #2 to read and evaluate the changes we made. At the same time, several tracked changes remain to fulfil PLOS One requirements that a version with tracked changes be included in the submission. | A clean manuscript file and a tracked changes version that is free of duplicated text have been uploaded for review. |
| **Additional suggestions to strengthen clarity and interpretation.** | | |
| *a. Maintain consistent language throughout the Abstract, Results narrative, and tables regarding which values are used for inference. If you state that conclusions are based on q values, ensure the narrative highlights adjusted results consistently and avoids mixing p based and q based interpretations.* | We appreciate this suggestion. The manuscript was revised to ensure that statistical inference is consistently based on FDR-adjusted q values, while both p values and q values are reported in tables for transparency. | The abstract, Results narrative, and tables were revised to ensure consistent interpretation based on q-values. |
| *b. Because the CCVD subgroup analyses involve small cell counts and multiple comparisons, please frame these findings explicitly as exploratory, and consider reporting effect sizes or risk differences alongside significance testing to support practical interpretation.* | We thank Reviewer #2 for this suggestion. The Discussion section now clarifies that subgroup analyses within the CCVD group should be interpreted as exploratory, given the smaller sample size and multiple comparisons. | The Discussion section has been updated to state that subgroup findings are exploratory and hypothesis-generating.  In lines 286-288:  ‘However, given the relatively small subgroup sample size and multiple comparisons examined, these findings should be interpreted as exploratory and hypothesis-generating, and future studies with larger samples are needed to confirm these associations.’ |
| *Ethics and publication ethics. Ethical approvals are stated. I did not identify clear concerns about research ethics from the information provided. If any part of the dataset cannot be shared publicly due to privacy restrictions, this should be stated explicitly in the Data Availability section, along with the mechanism for controlled access.* | We confirm that the dataset shared in the repository has been fully de-identified and contains no personally identifiable information. | Data Availability section updated to clarify that the dataset is fully anonymised. A separate codebook file has been uploaded on FigShare. |
