# Supplementary material for: Behavioural and emotional assessment of Malaysian adolescent boys with congenital colour vision deficiency and its association with socioeconomic factors
Source: PLoS One. 2026 Jul 23;21(7):e0353847. doi: 10.1371/journal.pone.0353847 (PMC13395452; doi:10.1371/journal.pone.0353847)
Supplement: Attachment — Submitted filename: Reviewer report for authors120426.docx [file pone.0353847.s009.docx]

## Structured reviewer responses

**1. Is the manuscript technically sound, and do the data support the conclusions?**
**Answer: Partly**

The study design is acceptable for the research question. The authors screened 2,879 boys, identified 44 with CCVD, matched them with 44 NCV controls, used validated color vision tests, and assessed behavioural outcomes using the Malay CBCL/4–18. They also state that FDR adjustment was applied, and they report effect sizes with confidence intervals for chi-square analyses.

However, the conclusions are not yet fully supported because key narrative interpretations do not consistently match the tabulated results. In Table 5, the manuscript states that lower parental education is associated with worse broad-band outcomes, yet the counts shown for Externalizing Problems and Total Problems appear worse in the tertiary group than in the secondary group. That contradiction must be resolved before the results can be trusted.

**2. Has the statistical analysis been performed appropriately and rigorously?**
**Answer: Partly**

The use of non-parametric comparisons after Shapiro-Wilk testing, followed by chi-square tests and FDR correction, is broadly reasonable for this sample and outcome structure.

That said, there are still reporting concerns: The manuscript claims significant broad-band differences for both Internalizing Problems and Externalizing Problems in the Results narrative and Table 3, yet the abstract mentions only Externalizing Problems. The socioeconomic subgroup interpretation is inconsistent with the displayed frequencies in Table 5, and the conclusion overstates income-related findings by naming domains not statistically significant in Table 6, including Attention Problems and Externalizing Problems. In Table 6, low income is significantly associated with Withdrawn, Anxious/Depressed, Thought Problems, and Aggressive Behaviour, but not with Attention Problems, Externalizing Problems, or Total Problems after FDR correction.

**3. Have the authors made all data underlying the findings fully available?**
**Answer: Yes, with a minor clarification request**

The submission materials now state that all data files, the codebook, and scoring documentation are available in Figshare, and a DOI is provided. That is a strong improvement and is consistent with the journal’s data-availability requirement.

I still suggest that the final manuscript text explicitly state whether the uploaded material contains raw CBCL item data, derived T-scores, grouping variables, and the exact coding used for the socioeconomic subgroup analyses, to make reanalysis straightforward.

**4. Is the manuscript presented in an intelligible fashion and written in standard English?**
**Answer: Yes, but minor editing is still required**

The paper is readable and mostly well organized. The introduction, methods, and discussion are logically structured. Still, a few language and presentation issues remain, including minor typographic errors, inconsistent wording, and table-to-text mismatches that create interpretive confusion. For example, “The CBCLwere distributed” needs correction, and several statements in the abstract, results, and conclusion need harmonization with the tables.

## Review comments to the authors

### Overall assessment

This manuscript examines behavioural and emotional outcomes in Malaysian adolescent boys with congenital colour vision deficiency and compares them with age- and class-matched peers with normal colour vision. The topic is worthwhile and underrepresented in the literature. The study has several strengths, including school-based ascertainment, clinically relevant vision testing, the use of a recognized behavioural assessment tool, and explicit ethics approval. The revised submission also improves transparency by adding a public data repository entry with DOI.

At the same time, the manuscript still needs one more substantive revision because some central interpretations are not internally consistent with the reported tables. These issues affect the validity of the conclusions and therefore must be corrected before the manuscript can be considered for acceptance.

### Main strengths

The study uses a clearly defined school-based comparative design and identifies CCVD through established color vision procedures rather than self-report.

The behavioural outcome assessment is clinically recognizable and standardized, using the Malay CBCL/4–18 with clear threshold definitions for borderline and clinical ranges.

The authors account for multiple testing using the Benjamini-Hochberg FDR procedure and report effect sizes with confidence intervals for chi-square analyses.

The manuscript addresses an important practical issue, namely the low parental awareness of CCVD and the implications of delayed detection. Only 12 of 44 mothers in the CCVD group were aware of the child's color-related difficulty.

### Major concerns

**1. The interpretation of parental education in Table 5 appears contradictory and may be incorrect.**
Your text states that lower parental education is significantly associated with worse behavioural outcomes. However, the table shows that for Externalizing Problems and Total Problems, the tertiary-education subgroup appears to have a markedly higher proportion in the borderline clinical plus clinical category than the secondary-education subgroup. Unless the group labels or column ordering are wrong, the current narrative interpretation is opposite to the table. This must be checked carefully against the original dataset and corrected throughout the Results, Discussion, Abstract, and Conclusion.

**2. The conclusion overstates the income findings relative to Table 6.**
In the conclusion, you state that low household income is associated with higher scores in domains such as Attention Problems and Externalizing Problems. Table 6 does not support that statement after FDR correction. The statistically significant income-associated domains are Withdrawn, Anxious/Depressed, Thought Problems, and Aggressive Behaviour. Attention Problems, Externalizing Problems, and Total Problems are not significant in Table 6. Please revise the conclusion so that it matches the corrected results exactly.

**3. The abstract and Results section are not fully harmonized regarding broad-band findings.**
In the Results text and Table 3, both Internalizing Problems and Externalizing Problems are reported as significantly higher in the CCVD group. The abstract, however, mentions only Externalizing Problems at the broad-band level. Please make the abstract, main Results, Discussion, and Conclusion fully consistent.

**4. Please distinguish more clearly between association and causation.**
The study is cross-sectional and based on parent-reported outcomes. Some phrases in the Discussion and Conclusion imply directional psychosocial effects of CCVD. The wording should remain strictly observational. Statements such as “CCVD may contribute to” or “is associated with” are acceptable. Stronger causal phrasing should be avoided unless explicitly framed as hypothesis-generating.

**5. The parental-awareness subgroup findings should be presented more cautiously.**
The awareness analysis is interesting, but only 12 parents were in the “aware” subgroup. Because all aware parents rated their adolescents in the normal range for all narrow-band scales, the possibility of small-cell instability or reporting bias is substantial. Your discussion already acknowledges that this is exploratory, and that caution should remain prominent in the abstract and conclusion if these findings are retained.

### Minor concerns and editorial points

Please correct minor typographic and grammatical issues, including “The CBCLwere distributed.”

Please ensure consistent use of British or American spelling throughout. At present, the manuscript mixes forms such as behavioural and behavior in references and descriptors.

Please clarify in the methods whether the socioeconomic cut points, especially the household income threshold, were prespecified or selected post hoc. The threshold is currently given as ≤ MYR4850 versus > MYR4850, but the rationale should be stated explicitly.

Please consider adding one concise sentence in the limitations section explaining that all 44 CCVD cases were deutan, which restricts generalizability to other forms of congenital color vision deficiency. Although you note that the D-15 may under-identify mild defects, the deutan-only composition is also important.

### Suggested decision

I recommend **minor revision**. The study is potentially suitable for publication, but only after the authors verify the subgroup analyses, correct the mismatches between tables and narrative text, and align the abstract and conclusion strictly with the reported statistics.
