# Supplementary material for: Behavioural and emotional assessment of Malaysian adolescent boys with congenital colour vision deficiency and its association with socioeconomic factors
Source: PLoS One. 2026 Jul 23;21(7):e0353847. doi: 10.1371/journal.pone.0353847 (PMC13395452; doi:10.1371/journal.pone.0353847)
Supplement: Attachment — Submitted filename: Response_to_Reviewers_auresp_4.docx [file pone.0353847.s011.docx]

**Title: Behavioural and Emotional Assessment of Malaysian Adolescent Boys with Congenital Colour Vision Deficiency and Its Association with Socioeconomic Factors**

We sincerely thank the reviewers for their careful evaluation of our manuscript and for providing constructive comments that have greatly improved the clarity, rigor, and overall quality of our work. We have carefully considered each point raised and revised the manuscript accordingly. Below, we provide a detailed, point‑by‑point response to all comments. Reviewer comments are listed in the left column, and our replies and corresponding corrections are presented in the right column.

| **Reviewer’s Comment** | **Authors’ Reply** |
| --- | --- |
| 1. ***Is the manuscript technically sound, and do the data support the conclusions?***   [R1]: In Table 5, the manuscript states that lower parental education is associated with worse broad-band outcomes, yet the counts shown for Externalizing Problems and Total Problems appear worse in the tertiary group than in the secondary group. That contradiction must be resolved before the results can be trusted.  [R2]: The manuscript must describe a technically sound piece of scientific research with data that supports the conclusions. Experiments must have been conducted rigorously, with appropriate controls, replication, and sample sizes. The conclusions must be drawn appropriately based on the data presented. | [R1]: We thank the reviewer for identifying this inconsistency. Upon re-evaluation of the data, we acknowledge that the original interpretation was inaccurate. Although the manuscript stated that lower parental education was associated with poorer broadband behavioural outcomes, the *proportions* of adolescents with borderline/clinical Externalising Problems and Total Problems were, in fact, higher among parents with tertiary education. The Results and Discussion sections have been revised to reflect the observed findings accurately.  The following was corrected and added in the Results section (lines 216-224):  *“These results indicate a significant relationship between parental education and behavioural outcomes among adolescents with CCVD. For* Externalising Problems*, seven adolescents whose parents had secondary education and six adolescents whose parents had tertiary education were classified within the borderline/clinical range. However, proportionally, the prevalence was higher in the tertiary education group (85.7%) than the secondary education group (18.9%) (p < .001). Similarly, for* Total Problems*, 14 adolescents whose parents had secondary education and six adolescents whose parents had tertiary education were classified within the borderline/clinical range, with a higher proportion observed among the tertiary education group (85.7%) than the secondary education group (37.8%).”*  In the Discussion section, the following was added (lines 353-359):  *“In contrast, higher proportions of* Externalising Problems *and* Total Problems *were observed among adolescents whose parents had tertiary education attainment. Given the small sample size (n=7) within the tertiary education subgroup, this finding should be interpreted cautiously and may reflect sampling imbalance or differences in parental awareness and reporting behaviours. In addition, parents with higher educational attainment may be more aware of behavioural and emotional concerns, potentially contributing to increased reporting of such difficulties”* |
| 2. ***Has the statistical analysis been performed appropriately and rigorously?***   [R1]: The manuscript claims significant broad-band differences for both Internalizing Problems and Externalizing Problems in the Results narrative and Table 3, yet the abstract mentions only Externalizing Problems.  The socioeconomic subgroup interpretation is inconsistent with the displayed frequencies in Table 5, and the conclusion overstates income-related findings by naming domains not statistically significant in Table 6, including Attention Problems and Externalizing Problems. In Table 6, low income is significantly associated with Withdrawn, Anxious/Depressed, Thought Problems, and Aggressive Behaviour, but not with Attention Problems, Externalizing Problems, or Total Problems after FDR correction.  [R2]: N/A | The abstract has been corrected to include Internalising Problems and Externalising Problems. It now reads (Abstract, page 1):  *“Adolescents with CCVD scored significantly higher on most CBCL/4-18 narrow-band scales (Anxious/Depressed, Withdrawn, Thought Problems, Attention Problems, Delinquent Behaviour, Aggressive Behaviour), broad-band scales (Internalising Problems and Externalising Problems), and Total Problems (all q<0.05).”*  Table 5 displays parental education level, whereas Table 6 displays parental monthly household income. We believe the Reviewer is referring to Table 6.  The interpretation of income-related findings and significant associated behavioural problems has been corrected (lines 228-236):  *“Significant associations were also identified between monthly household income (low vs. high) and behavioural problems among adolescents with CCVD (Table 6). Narrow-band scales showing significant associations included* Withdrawn, *χ²(1, N = 44) = 26.882, q=0.002, Cramér’s V =.782, 95% CI [.546,.951],* Anxious/Depressed, *χ²(1, N = 44) = 12.617, q=0.002, Cramér’s V =.535, 95% CI [.239,.795],* Thought Problems*, χ²(1, N = 44) = 26.882, q=0.002, Cramér’s V =.782, 95% CI [.546,.951], and* Aggressive Behaviour, *χ²(1, N = 44) = 12.617, q=0.002, Cramér’s V =.535, 95% CI [.239,.795]. In other words, there is a significant relationship between lower monthly household income and some behavioural difficulties among adolescents with CCVD.”* |
| 3. ***Have the authors made all data underlying the findings fully available?***   [R1]: I still suggest that the final manuscript text explicitly state whether the uploaded material contains raw CBCL item data, derived T-scores, grouping variables, and the exact coding used for the socioeconomic subgroup analyses, to make reanalysis straightforward.  [R2]: Yes | The data availability is mentioned in Plos One’s required Data Availability statement. It is also mentioned at the end of the manuscript (lines 421-423)  *“Data availability*  *All data are available without restrictions. The uploaded dataset contains raw CBCL item data, derived T-scores, and the exact coding used for all sub-group analyses.”* |
| ***4. Is the manuscript presented in an intelligible fashion and written in standard English?***  [R1]: Yes, but minor editing is still required.  The paper is readable and mostly well organized. The introduction, methods, and discussion are logically structured. Still, a few language and presentation issues remain, including minor typographic errors, inconsistent wording, and table-to-text mismatches that create interpretive confusion. For example, “The CBCLwere distributed” needs correction, and several statements in the abstract, results, and conclusion need harmonization with the tables.  [R2]: Yes | Language and presentation issues, typographic errors, inconsistent wording, and table-to-text mismatches have been corrected. |
| **Major Concerns** | |
| **[R1]: 1**. **The interpretation of parental education in Table 5 appears contradictory and may be incorrect.**  Your text states that lower parental education is significantly associated with worse behavioural outcomes. However, the table shows that for Externalizing Problems and Total Problems, the tertiary-education subgroup appears to have a markedly higher proportion in the borderline clinical plus clinical category than the secondary-education subgroup. Unless the group labels or column ordering are wrong, the current narrative interpretation is opposite to the table. This must be checked carefully against the original dataset and corrected throughout the Results, Discussion, Abstract, and Conclusion**.** | [R1]: We thank the reviewer for identifying this inconsistency. Upon re-evaluation of the data, we acknowledge that the original interpretation was inaccurate. Although the manuscript stated that lower parental education was associated with poorer broadband behavioural outcomes, the proportions of adolescents with borderline/clinical Externalising Problems and Total Problems were, in fact, higher among parents with tertiary education. The Results and Discussion sections have been revised accordingly to reflect the observed findings accurately.  The following was corrected and added in the Results section (lines 216-224):  *“These results indicate a significant relationship between parental education and behavioural outcomes among adolescents with CCVD. For* Externalising Problems*, seven adolescents whose parents had secondary education and six adolescents whose parents had tertiary education were classified within the borderline/clinical range. However, proportionally, the prevalence was higher in the tertiary education group (85.7%) than the secondary education group (18.9%) (p < .001). Similarly, for* Total Problems*, 14 adolescents whose parents had secondary education and six adolescents whose parents had tertiary education were classified within the borderline/clinical range, with a higher proportion observed among the tertiary education group (85.7%) than the secondary education group (37.8%).”*  In the Discussion section, the following was added (lines 353-359):  *“In contrast, higher proportions of* Externalising Problems *and* Total Problems *were observed among adolescents whose parents had tertiary education attainment. Given the small sample size (n=7) within the tertiary education subgroup, this finding should be interpreted cautiously and may reflect sampling imbalance or differences in parental awareness and reporting behaviours. In addition, parents with higher educational attainment may be more aware of behavioural and emotional concerns, potentially contributing to increased reporting of such difficulties”*  The Abstract has been corrected, and the following has been added:  *“Adolescents with CCVD scored significantly higher on most CBCL/4-18 narrow-band scales (*Anxious/Depressed, Withdrawn, Thought Problems, Attention Problems, Delinquent Behaviour, Aggressive Behaviour*), broad-band scales (*Internalising Problems *and* Externalising Problems*), and* Total Problems *(all q<0.05).”*  The following has also been added in the Abstract:  *“Among boys with CCVD, those whose parents had tertiary education demonstrated higher proportions of behavioural difficulties in the* Externalising Problems *and* Total Problems *domains; however, these findings should be interpreted with caution due to the small subgroup size. Lower household income was significantly associated with* Withdrawn, Anxious/Depressed, Thought Problems*, and* Aggressive Behaviour *scores (all q < 0.05). In conclusion, the results indicate that psychosocial and environmental factors may influence behavioural outcomes in children with CCVD, highlighting the importance of early identification and supportive interventions.”*  The Conclusion has been corrected, and the following has been added.  Lines 401-404:  *“Elevated scores were observed on multiple CBCL/4–18 narrow-band scales, particularly* Anxious/Depressed, Withdrawn, Thought Problems, Attention Problems, Delinquent Behaviour*, and* Aggressive Behaviour*, as well as on broad-band scales (*Internalising Problems *and* Externalising Problems*) and* Total Problems*.”*  Lines 406-412:  *“Adolescent boys from lower-income households demonstrated substantially higher proportions of behavioural problems like* Withdrawn, Anxious/Depressed, Thought Problems *and* Aggressive Behaviour*. In contrast, higher proportions of* Externalising Problems *and* Total Problems *were observed among adolescents whose parents had tertiary education attainment. Given the small sample size within the tertiary education subgroup, this finding should be interpreted cautiously and may reflect sampling imbalance or differences in parental awareness and reporting behaviours.”* |
| **[R1]: 2. The conclusion overstates the income findings relative to Table 6.**  In the conclusion, you state that low household income is associated with higher scores in domains such as Attention Problems and Externalizing Problems. Table 6 does not support that statement after FDR correction. The statistically significant income-associated domains are Withdrawn, Anxious/Depressed, Thought Problems, and Aggressive Behaviour. Attention Problems, Externalizing Problems, and Total Problems are not significant in Table 6. Please revise the conclusion so that it matches the corrected results exactly. | This has been corrected in the Conclusion section as follows (lines 406-408):  *“Adolescent boys from lower-income households demonstrated substantially higher proportions of behavioural problems like Withdrawn, Anxious/Depressed, Thought Problems and Aggressive Behaviour.”* |
| **[R1]: 3. The abstract and Results section are not fully harmonized regarding broad-band findings.** In the Results text and Table 3, both Internalizing Problems and Externalizing Problems are reported as significantly higher in the CCVD group. The abstract, however, mentions only Externalizing Problems at the broad-band level. Please make the abstract, main Results, Discussion, and Conclusion fully consistent. | This has been corrected in the Abstract, Results, Discussion and Conclusion sections.  The Abstract has been corrected to include Internalising Problems and Externalising Problems. It now reads (Abstract, page 1):  *“Adolescents with CCVD scored significantly higher on most CBCL/4-18 narrow-band scales (*Anxious/Depressed, Withdrawn, Thought Problems, Attention Problems, Delinquent Behaviour, Aggressive Behaviour*), broad-band scales (*Internalising Problems *and* Externalising Problems*), and* Total Problems *(all q<0.05).”*  In the main Results (lines 184-192):  *“Significant differences were found for both broad-band scales, where adolescent boys with CCVD scored significantly higher on* Internalising Problems *and* Externalising Problems *compared with those in the NCV group (both q < 0.05). Among the narrow-band scales, the CCVD group scored significantly higher on six scales:* Withdrawn, Anxious/Depressed, Thought Problems, Attention Problems, Delinquent Behaviour, *and* Aggressive Behaviour *(all q < 0.05).*  *Additionally, adolescent boys with CCVD scored significantly higher for* Total Problems *than the NCV group (U=600.00, q=0.002).”*  In the Discussion (lines 258-261):  *“The present study revealed that adolescent boys with CCVD exhibited significantly higher scores on several CBCL/4-18 narrow-band scales, including* Anxious/Depressed, Withdrawn, Thought Problems, Attention Problems, Delinquent Behaviour, *and* Aggressive Behaviour.”  In lines 266-269:  *“The concurrent elevation in* Internalising Problems, Externalising Problems *and* Total Problems *further implies that the impact of CCVD may extend beyond colour-related functional limitations to include wider psychosocial and behavioural functioning.”*  In the Conclusion (lines 401-404):  *Elevated scores were observed on multiple CBCL/4–18 narrow-band scales, particularly Anxious/Depressed, Withdrawn, Thought Problems, Attention Problems, Delinquent Behaviour, and Aggressive Behaviour, as well as on broad-band scales (Internalising Problems and Externalising Problems) and Total Problems.* |
| **[R1]: 4. Please distinguish more clearly between association and causation.** The study is cross-sectional and based on parent-reported outcomes. Some phrases in the Discussion and Conclusion imply directional psychosocial effects of CCVD. The wording should remain strictly observational. Statements such as “CCVD may contribute to” or “is associated with” are acceptable. Stronger causal phrasing should be avoided unless explicitly framed as hypothesis-generating. | Sentences in the Discussion have been rephrased as below (lines 262-271):  *“The findings indicate that adolescent boys with CCVD experience a broader pattern of emotional and behavioural difficulties compared with their peers with normal colour vision. The strongest association between CCVD and* Attention Problems *suggests that attentional and behavioural regulation difficulties may represent particularly important challenges in this population. The concurrent elevation in* Internalising Problems, Externalising Problems *and* Total Problems *further implies that the impact of CCVD may extend beyond colour-related functional limitations to include wider psychosocial and behavioural functioning. Overall, these results support the possibility that CCVD is associated with increased vulnerability to emotional, attentional, and behavioural difficulties during adolescence.”*  In lines 278-285:  *“Accordingly, while age-related factors may have influenced CBCL scores overall, the study’s findings suggest that the elevated behavioural and emotional problem scores observed among adolescent boys with CCVD are unlikely to be attributable solely to normal adolescent developmental or pubertal changes. Because participants were compared with age-matched peers with normal colour vision, the observed group differences more likely reflect factors associated with CCVD itself rather than general age-related developmental influences. This supports the interpretation that CCVD may contribute to increased emotional and behavioural vulnerability during adolescence.”*  In lines 338-343:  *“The present study found that adolescents with CCVD from lower-income households demonstrated substantially higher proportions of behavioural problems like* Withdrawn, Anxious/Depressed, Thought Problems *and* Aggressive Behaviour. *This finding is consistent with previous literature suggesting that financial constraints may contribute to psychosocial stressors, reduced access to support resources, and increased emotional and behavioural difficulties among children and adolescents (28).”*  In lines 353-359:  *“In contrast, higher proportions of Externalising Problems and Total Problems were observed among adolescents whose parents had tertiary education attainment. Given the small sample size (n=7) within the tertiary education subgroup, this finding should be interpreted cautiously and may reflect sampling imbalance or differences in parental awareness and reporting behaviours. In addition, parents with higher educational attainment may be more aware of behavioural and emotional concerns, potentially contributing to increased reporting of such difficulties.”*  Sentences in the Conclusion have been rephrased as below (lines 406-412):  *Adolescent boys from lower-income households demonstrated substantially higher proportions of behavioural problems like* Withdrawn, Anxious/Depressed, Thought Problems *and* Aggressive Behaviour*. In contrast, higher proportions of* Externalising Problems *and* Total Problems *were observed among adolescents whose parents had tertiary education attainment. Given the small sample size within the tertiary education subgroup, this finding should be interpreted cautiously and may reflect sampling imbalance or differences in parental awareness and reporting behaviours.* |
| **[R1]: 5. The parental-awareness subgroup findings should be presented more cautiously.** The awareness analysis is interesting, but only 12 parents were in the “aware” subgroup. Because all aware parents rated their adolescents in the normal range for all narrow-band scales, the possibility of small-cell instability or reporting bias is substantial. Your discussion already acknowledges that this is exploratory, and that caution should remain prominent in the abstract and conclusion if these findings are retained. | We agree that the parental-awareness subgroup findings should be interpreted cautiously due to the small number of aware parents (n = 12) and the potential for reporting bias or small-cell instability. The Abstract and Conclusion have been revised to emphasise that these findings are exploratory.  In the Abstract:  *“….however, these findings should be interpreted with caution due to the small subgroup size.”*  In the Conclusion (lines 410-412):  *“Given the small sample size within the tertiary education subgroup, this finding should be interpreted cautiously and may reflect sampling imbalance or differences in parental awareness and reporting behaviours.”* |
| **[R2]:** My main concern is that the narrative interpretation of the socioeconomic subgroup analyses does not appear to match the tabulated data. In the Results text, the manuscript states that lower parental education is significantly related to worse behavioural outcomes among adolescents with CCVD. However, in Table 5, the broad-band frequencies shown for Externalizing Problems and Total Problems appear higher in the tertiary-education subgroup than in the secondary-education subgroup. The Results text on parental education, the counts in Table 5, the Discussion, the Abstract, and the Conclusion therefore do not seem fully aligned. This issue must be checked carefully against the source dataset and corrected throughout the manuscript. | This has been corrected.  The interpretation of income-related findings and significant associated behavioural problems has been corrected in Results.  On household income, lines 228-236:  *“Significant associations were also identified between monthly household income (low vs. high) and behavioural problems among adolescents with CCVD (Table 6). Narrow-band scales showing significant associations included* Withdrawn, *χ²(1, N = 44) = 26.882, q=0.002, Cramér’s V =.782, 95% CI [.546,.951],* Anxious/Depressed, *χ²(1, N = 44) = 12.617, q=0.002, Cramér’s V =.535, 95% CI [.239,.795],* Thought Problems*, χ²(1, N = 44) = 26.882, q=0.002, Cramér’s V =.782, 95% CI [.546,.951], and* Aggressive Behaviour, *χ²(1, N = 44) = 12.617, q=0.002, Cramér’s V =.535, 95% CI [.239,.795]. In other words, there is a significant relationship between lower monthly household income and some behavioural difficulties among adolescents with CCVD.”*  On parental education, lines 211-224:  *“Among adolescent boys with CCVD, there were statistically significant relationships between their parental education level and behavioural outcomes on broad-band CBCL scales (Table 5); specifically for broad-band scale:* Externalising Problems*, χ²(1, N = 44) = 12.617, p < 0.001, q = 0.002, Cramér’s V =0.535, 95% CI [0.220,.0787] and Total Problems, χ²(1, N = 44) = 5.442, q=0.02, Cramér’s V =.352, 95% CI [.066,.594] that showed strong to moderate effect sizes. These results indicate a significant relationship between parental education and behavioural outcomes among adolescents with CCVD. For* Externalising Problems*, seven adolescents whose parents had secondary education and six adolescents whose parents had tertiary education were classified within the borderline/clinical range. However, proportionally, the prevalence was higher in the tertiary education group (85.7%) than the secondary education group (18.9%) (p < .001). Similarly, for* Total Problems*, 14 adolescents whose parents had secondary education and six adolescents whose parents had tertiary education were classified within the borderline/clinical range, with a higher proportion observed among the tertiary education group (85.7%) than the secondary education group (37.8%).”*  In the Discussion section, the following was added:  On household income, lines 338-343:  *“The present study found that adolescents with CCVD from lower-income households demonstrated substantially higher proportions of behavioural problems like* Withdrawn, Anxious/Depressed, Thought Problems *and* Aggressive Behaviour*. This finding is consistent with previous literature suggesting that financial constraints may contribute to psychosocial stressors, reduced access to support resources, and increased emotional and behavioural difficulties among children and adolescents (28)”*  On parental education, lines 353-359:  *“In contrast, higher proportions of* Externalising Problems *and* Total Problems *were observed among adolescents whose parents had tertiary education attainment. Given the small sample size (n=7) within the tertiary education subgroup, this finding should be interpreted cautiously and may reflect sampling imbalance or differences in parental awareness and reporting behaviours. In addition, parents with higher educational attainment may be more aware of behavioural and emotional concerns, potentially contributing to increased reporting of such difficulties”*  In the Conclusion section (lines 406-410):  *“Adolescent boys from lower-income households demonstrated substantially higher proportions of behavioural problems like* Withdrawn, Anxious/Depressed, Thought Problems *and* Aggressive Behaviour. *In contrast, higher proportions of* Externalising Problems *and* Total Problems *were observed among adolescents whose parents had tertiary education attainment.”* |
| **[R2]:** A related issue affects the household-income interpretation. Table 6 supports significant associations for Withdrawn, Anxious/Depressed, Thought Problems, and Aggressive Behaviour, but not for Attention Problems, Externalizing Problems, or Total Problems after FDR correction. Despite this, the Conclusion states that low household income is significantly associated with higher scores in domains such as Attention Problems and Externalizing Problems. That statement should be revised so that it matches the reported q values exactly. | This has been corrected. Please see above. |
| **[R2]:** There is also an inconsistency between the abstract and the main Results regarding the broad-band findings. In the Results section and Table 3, both Internalizing Problems and Externalizing Problems are reported as significantly higher in the CCVD group. In the abstract, however, only Externalizing Problems is highlighted at the broad-band level. Please harmonize the abstract, Results, Discussion, and Conclusion. | This has been corrected in the Abstract, Results, Discussion, and Conclusion sections. |
| **[R2]:** On statistical reporting, the general analytical approach is reasonable. The manuscript describes normality assessment with Shapiro-Wilk, non-parametric group comparisons with Mann-Whitney U, chi-square tests for categorized T-scores, and FDR adjustment with inference based on q values. Still, because the subgroup findings are central to the paper’s interpretation, the authors should re-check the subgroup tables and all corresponding narrative statements carefully before any final decision. | All data and inferences based on q values have been rechecked and correspond to narrative statements. |
| **[R1 & R2]: Minor concerns and editorial points** | |
| - Please correct minor typographic and grammatical issues, including “The CBCLwere distributed.” - Please ensure consistent use of British or American spelling throughout. At present, the manuscript mixes forms such as behavioural and behavior in references and descriptors. - Please clarify in the methods whether the socioeconomic cut points, especially the household income threshold, were prespecified or selected post hoc. The threshold is currently given as ≤ MYR4850 versus > MYR4850, but the rationale should be stated explicitly. - Please consider adding one concise sentence in the limitations section explaining that all 44 CCVD cases were deutan, which restricts generalizability to other forms of congenital color vision deficiency. Although you note that the D-15 may under-identify mild defects, the deutan-only composition is also important. | Typographic and grammar issues have been corrected.  The spellings have been corrected to follow British English spelling.  This sentence has been added to the Statistical Analysis section (lines 149-150):  *“The household income cut-off of MYR 4,850 was prespecified based on national income benchmarks to differentiate lower- and higher-income groups.”*  This sentence has been added to the Discussion section under limitation (lines 395-398):  *“In addition, all CCVD cases identified in the present study were classified as deutan defects, which may limit the generalisability of the findings to other forms of congenital colour vision deficiency, particularly protan defects.”* |
