# Supplementary material for: Behavioural and emotional assessment of Malaysian adolescent boys with congenital colour vision deficiency and its association with socioeconomic factors
Source: PLoS One. 2026 Jul 23;21(7):e0353847. doi: 10.1371/journal.pone.0353847 (PMC13395452; doi:10.1371/journal.pone.0353847)
Supplement: Attachment — Submitted filename: Rev.5_Authors Reply_PlosOne_20June.docx [file pone.0353847.s012.docx]

**Behavioural and Emotional Issues in Malaysian Adolescent Boys with Congenital Colour Vision Deficiency and Their Association with Socioeconomic Factors. [PONE-D-25-42304R3]**

**Response to Reviewers’ Comments (5^th^ Review):**

| **Reviewer Comment** | **Author Response** |
| --- | --- |
| **1. Please verify the confidence interval reported for Cramér's V in Table 5 (Externalising Problems), as the reported interval appears inconsistent and may contain a typographical error.** | Typographical error.  We have checked and corrected the decimal points for all figures throughout the manuscript. Specifically for this comment, the revised CI in the main text now reads: **Cramér’s V = 0.535, 95% CI [0.220, 0.787]** |
| **2. Please review the Discussion and Conclusion sections to ensure that all interpretations remain strictly associative rather than causal, given the cross-sectional design of the study.** | Revised Discussion and Conclusion sections to ensure strictly associative language (e.g., “**associated with**,” “**linked to**,” “**observed in**”).  All **causal wording has been removed or rephrased.**  In **Discussion, page 18, lines 262-264:**  *“The findings suggest that adolescent boys with CCVD may experience a broader pattern of emotional and behavioural difficulties compared with their peers with normal colour vision.”*  **Page 18, lines 266-269:**  *“The concurrent elevation in Internalising Problems, Externalising Problems, and Total Problems further indicates that CCVD is associated with broader psychosocial and behavioural outcomes beyond colour-related functional limitations.”*  **Page 18, lines 271-274:**  *“These findings are consistent with previous reports documenting psychosocial and emotional challenges faced by individuals with CCVD (7,20) and further support an association between CCVD and increased psychosocial and emotional difficulties during development.”*  **Page 18, lines 284-285:**    *“This further supports an association between CCVD and increased emotional and behavioural vulnerability during adolescence.”*  **Page 21, lines 349-351**:  *“Low socioeconomic status is associated with worsened emotional and behavioural outcomes, which may not be as apparent in adolescents with CCVD from higher socioeconomic status groups.”*  In the **Conclusion** section**, page 23, lines 400-401**:  *“Adolescent boys with CCVD were reported to have significantly higher behavioural and emotional problems compared to peers with NCV.”*  **Page 24, lines 413-416:**  *“Routine school-based colour vision screening, coupled with early behavioural and emotional assessment and targeted educational support, may help support adolescents and reduce potential long-term psychosocial challenges among adolescents with CCVD.”* |
| **3. Please perform a final consistency check between the tables and the corresponding narrative descriptions throughout the ms.** | We have conducted a full cross-check between the tables and their corresponding texts.  We have corrected all **typographical errors, ensured consistent table numbering, and aligned all narrative descriptions with reported table values**. |
| **4. Please verify that all reported confidence intervals, effect sizes, and statistical values are presented accurately and consistently.** | We have rechecked all statistical outputs. The standardised formatting is as follows:  • CI format: **leading zero** **[0.xxx, 0.xxx]**  • p-values: **leading zero (e.g., p = 0.006)**  • **χ² notation unified** |
| **5. Please conduct a final proofreading review to correct any remaining typographical, formatting, or presentation issues. I look forward to receiving the revised version.** | We have completed comprehensive proofreading.  We have corrected **grammar, improved sentence clarity, fixed spacing issues, and ensured consistent terminology (e.g., “Borderline Clinical/Clinical”**). |
