# Supplementary material for: Behavioural and emotional assessment of Malaysian adolescent boys with congenital colour vision deficiency and its association with socioeconomic factors
Source: PLoS One. 2026 Jul 23;21(7):e0353847. doi: 10.1371/journal.pone.0353847 (PMC13395452; doi:10.1371/journal.pone.0353847)
Supplement: Peer review history

# Peer review: Decision Letter 0

**DOI:** 10.1371/journal.pone.0353847.r001

**Authors:** Thiago Fernandes

2 Dec 2025

PONE-D-25-42304

Behavioural and emotional issues in Malaysian adolescent boys with congenital colour vision deficiency and their association with socioeconomic factors

PLOS ONE

Dear Dr. Hairol,

Thank you for submitting your manuscript to PLOS ONE. After careful consideration, we feel that it has merit but does not fully meet PLOS ONE’s publication criteria as it currently stands. Therefore, we invite you to submit a revised version of the manuscript that addresses the points raised during the review process.

==============================

Thank you for your valuable submission. Please note that the reviewers recognised the merit of your study, however, several important concerns were raised that will need to be addressed. Please respond to all comments and highlight them in the revised ms.

Wishing you success with the study.

==============================

Please submit your revised manuscript by Jan 16 2026 11:59PM. If you will need more time than this to complete your revisions, please reply to this message or contact the journal office at plosone@plos.org. When you're ready to submit your revision, log on to https://www.editorialmanager.com/pone/ and select the 'Submissions Needing Revision' folder to locate your manuscript file.

Please include the following items when submitting your revised manuscript:

- A rebuttal letter that responds to each point raised by the academic editor and reviewer(s). You should upload this letter as a separate file labeled 'Response to Reviewers'.
- A marked-up copy of your manuscript that highlights changes made to the original version. You should upload this as a separate file labeled 'Revised Manuscript with Track Changes'.
- An unmarked version of your revised paper without tracked changes. You should upload this as a separate file labeled 'Manuscript'.

If you would like to make changes to your financial disclosure, please include your updated statement in your cover letter. Guidelines for resubmitting your figure files are available below the reviewer comments at the end of this letter.

If applicable, we recommend that you deposit your laboratory protocols in protocols.io to enhance the reproducibility of your results. Protocols.io assigns your protocol its own identifier (DOI) so that it can be cited independently in the future. For instructions see: https://journals.plos.org/plosone/s/submission-guidelines#loc-laboratory-protocols. Additionally, PLOS ONE offers an option for publishing peer-reviewed Lab Protocol articles, which describe protocols hosted on protocols.io. Read more information on sharing protocols at https://plos.org/protocols?utm_medium=editorial-email&utm_source=authorletters&utm_campaign=protocols.

We look forward to receiving your revised manuscript.

Kind regards,

Thiago P. Fernandes, PhD

Academic Editor

PLOS ONE

**Journal Requirements:**

1. When submitting your revision, we need you to address these additional requirements.

Please ensure that your manuscript meets PLOS ONE's style requirements, including those for file naming. The PLOS ONE style templates can be found at

https://journals.plos.org/plosone/s/file?id=wjVg/PLOSOne_formatting_sample_main_body.pdf and

https://journals.plos.org/plosone/s/file?id=ba62/PLOSOne_formatting_sample_title_authors_affiliations.pdf

2. Please note that your Data Availability Statement is currently missing the repository name. If your manuscript is accepted for publication, you will be asked to provide these details on a very short timeline. We therefore suggest that you provide this information now, though we will not hold up the peer review process if you are unable.

3. If the reviewer comments include a recommendation to cite specific previously published works, please review and evaluate these publications to determine whether they are relevant and should be cited. There is no requirement to cite these works unless the editor has indicated otherwise.

[Note: HTML markup is below. Please do not edit.]

Reviewers' comments:

Reviewer's Responses to Questions

**Comments to the Author**

1. Is the manuscript technically sound, and do the data support the conclusions?

The manuscript must describe a technically sound piece of scientific research with data that supports the conclusions. Experiments must have been conducted rigorously, with appropriate controls, replication, and sample sizes. The conclusions must be drawn appropriately based on the data presented.

Reviewer #1: Yes

Reviewer #2: Yes

**********

2. Has the statistical analysis been performed appropriately and rigorously?

Reviewer #1: No

Reviewer #2: Yes

**********

3. Have the authors made all data underlying the findings in their manuscript fully available?

The PLOS Data policy requires authors to make all data underlying the findings described in their manuscript fully available without restriction, with rare exception (please refer to the Data Availability Statement in the manuscript PDF file). The data should be provided as part of the manuscript or its supporting information, or deposited to a public repository. For example, in addition to summary statistics, the data points behind means, medians and variance measures should be available. If there are restrictions on publicly sharing data—e.g. participant privacy or use of data from a third party—those must be specified.

Reviewer #1: No

Reviewer #2: Yes

**********

4. Is the manuscript presented in an intelligible fashion and written in standard English?

PLOS ONE does not copyedit accepted manuscripts, so the language in submitted articles must be clear, correct, and unambiguous. Any typographical or grammatical errors should be corrected at revision, so please note any specific errors here.

Reviewer #1: Yes

Reviewer #2: Yes

**********

5. Review Comments to the Author

Please use the space provided to explain your answers to the questions above. You may also include additional comments for the author, including concerns about dual publication, research ethics, or publication ethics. (Please upload your review as an attachment if it exceeds 20,000 characters)

**Reviewer #1:** Thank you for your research initiative on adolescent boys with congenital colour vision deficiency (CCVD). I have a few suggestions to further strengthen your findings. Has similar work been conducted previously on CCVD? Please consider adding a few more references. I also believe there is an opportunity to review and potentially revise the title.

A major table demonstrating CBCL/4-18 T scores alongside socioeconomic factors was not included in your file. Please upload your data as a supplementary file for better clarification. I have a query regarding the study design: why was this not conducted as a case-control study? Since the scores for Internalizing, Externalizing, and Total Problems are interdependent and derived from the narrow-band scales, the results should be adjusted using an appropriate statistical test to determine their actual significance.

Please check the attached file for other modifications.

**Reviewer #2:** This is a technically sound cross-sectional case–control study comparing behavioural and emotional problems in adolescent boys with CCVD versus age-/class-matched peers with normal colour vision. Diagnostic confirmation (Ishihara + Farnsworth D15), validated outcomes (CBCL/4–18 Malay), and appropriate nonparametric and categorical statistics make the analysis rigorous. Findings (elevated narrow-band scales including Anxious/Depressed, Withdrawn, Thought Problems, Attention Problems, Delinquent, Aggressive; plus higher Externalizing and Total Problems) are reported transparently and support the conclusions.

Major strengths

1. Clear design and recruitment across 10 schools with matching by age and class; sample size rationale is given.

2. Rigorous phenotyping (Ishihara under controlled luminance and D15 classification with defined error criteria).

3. Validated outcomes & scoring with clinical/borderline thresholds described.

4. Appropriate statistics with effect sizes, and results that align with conclusions (including socioeconomic associations within the CCVD subgroup).

5. Ethics approvals and open data comply with PLOS policy.

Suggestions for minor revision (clarifications/editorial)

Multiple comparisons: You test many CBCL scales. Please add a brief statement acknowledging no formal multiplicity adjustment (or provide justification if treating this as an exploratory multi-endpoint study). Consider adding confidence intervals for key effect sizes to aid interpretation.

Power justification: You state a target of n=44 per group from prevalence considerations; please add a sentence on the anticipated effect size and α used to deem this sample size adequate.

Reporter/source of outcome: Outcomes rely solely on parent (mother) report. Add a line in Limitations about possible reporting bias and the value of multi-informant data (e.g., youth self-report/teacher ratings) in future work—this is already hinted but could be made explicit.

Phenotyping sensitivity: Note that D15 primarily detects moderate–severe defects; very mild CCVD may be under-detected. You mention criteria—just add a succinct limitation sentence to signal awareness.

Editorial polish:

In the ethics statement, there is a small mixed-language connector (“dan the Department of Education…”)—please correct to “and the Department…”.

Consider trimming small redundancies in the Introduction where impacts of CCVD on schooling are reiterated to improve flow.

Conclusion & recommendation: Your results are consistent, methods are sound, and conclusions are appropriately circumscribed for a cross-sectional study. I recommend Minor Revision to incorporate the clarifications above.

**********

6. PLOS authors have the option to publish the peer review history of their article (what does this mean?). If published, this will include your full peer review and any attached files.

If you choose “no”, your identity will remain anonymous but your review may still be made public.

**Do you want your identity to be public for this peer review?**  For information about this choice, including consent withdrawal, please see our Privacy Policy.

Reviewer #1: **Yes:** Syeda Humaida Hasan

Reviewer #2: No

**********

[NOTE: If reviewer comments were submitted as an attachment file, they will be attached to this email and accessible via the submission site. Please log into your account, locate the manuscript record, and check for the action link "View Attachments". If this link does not appear, there are no attachment files.]

While revising your submission, please upload your figure files to the Preflight Analysis and Conversion Engine (PACE) digital diagnostic tool, https://pacev2.apexcovantage.com/. PACE helps ensure that figures meet PLOS requirements. To use PACE, you must first register as a user. Registration is free. Then, login and navigate to the UPLOAD tab, where you will find detailed instructions on how to use the tool. If you encounter any issues or have any questions when using PACE, please email PLOS at figures@plos.org. Please note that Supporting Information files do not need this step.

Attachment Submitted filename: PLOS Review on CCVD.docx

Attachment Submitted filename: REVIEW-Behavioural-091025.docx

# Author comment: Author response to Decision Letter 1

**DOI:** 10.1371/journal.pone.0353847.r002

1 Jan 2026

We sincerely thank the Reviewers for their careful evaluation of our manuscript. All reviewer comments have been addressed in the uploaded "Response to Reviewers" file. Both reviewers have provided their comments within the email from the Editor and in separate Word files. As such, we have structured our replies and feedback accordingly

Attachment Submitted filename: Response to Reviewers.docx

# Peer review: Decision Letter 1

**DOI:** 10.1371/journal.pone.0353847.r003

**Authors:** Thiago Fernandes

19 Jan 2026

PONE-D-25-42304R1

Behavioural and Emotional Assessment of Malaysian Adolescent Boys with Congenital Colour Vision Deficiency and its Association with Socioeconomic Factors.

PLOS One

Dear Dr. Hairol,

Thank you for submitting your manuscript to PLOS ONE. After careful consideration, we feel that it has merit but does not fully meet PLOS ONE’s publication criteria as it currently stands. Therefore, we invite you to submit a revised version of the manuscript that addresses the points raised during the review process.

==============================**l**

Please respond to all comments and highlight them in the revised ms.

==============================

Please submit your revised manuscript by Mar 05 2026 11:59PM. If you will need more time than this to complete your revisions, please reply to this message or contact the journal office at plosone@plos.org. When you're ready to submit your revision, log on to https://www.editorialmanager.com/pone/ and select the 'Submissions Needing Revision' folder to locate your manuscript file.

Please include the following items when submitting your revised manuscript:

- A letter that responds to each point raised by the academic editor and reviewer(s). You should upload this letter as a separate file labeled 'Response to Reviewers'.
- A marked-up copy of your manuscript that highlights changes made to the original version. You should upload this as a separate file labeled 'Revised Manuscript with Track Changes'.
- An unmarked version of your revised paper without tracked changes. You should upload this as a separate file labeled 'Manuscript'.

-->If you would like to make changes to your financial disclosure, please include your updated statement in your cover letter. Guidelines for resubmitting your figure files are available below the reviewer comments at the end of this letter.

If applicable, we recommend that you deposit your laboratory protocols in protocols.io to enhance the reproducibility of your results. Protocols.io assigns your protocol its own identifier (DOI) so that it can be cited independently in the future. For instructions see: https://journals.plos.org/plosone/s/submission-guidelines#loc-laboratory-protocols. Additionally, PLOS ONE offers an option for publishing peer-reviewed Lab Protocol articles, which describe protocols hosted on protocols.io. Read more information on sharing protocols at https://plos.org/protocols?utm_medium=editorial-email&utm_source=authorletters&utm_campaign=protocols.

We look forward to receiving your revised manuscript.

Kind regards,

Thiago P. Fernandes, PhD

Academic Editor

PLOS One

Journal Requirements:

1. If the reviewer comments include a recommendation to cite specific previously published works, please review and evaluate these publications to determine whether they are relevant and should be cited. There is no requirement to cite these works unless the editor has indicated otherwise.

2. Please review your reference list to ensure that it is complete and correct. If you have cited papers that have been retracted, please include the rationale for doing so in the manuscript text, or remove these references and replace them with relevant current references. Any changes to the reference list should be mentioned in the rebuttal letter that accompanies your revised manuscript. If you need to cite a retracted article, indicate the article’s retracted status in the References list and also include a citation and full reference for the retraction notice.

[Note: HTML markup is below. Please do not edit.]

Reviewers' comments:

Reviewer's Responses to Questions

**Comments to the Author**

1. If the authors have adequately addressed your comments raised in a previous round of review and you feel that this manuscript is now acceptable for publication, you may indicate that here to bypass the “Comments to the Author” section, enter your conflict of interest statement in the “Confidential to Editor” section, and submit your "Accept" recommendation.

Reviewer #1: All comments have been addressed

Reviewer #2: (No Response)

**********

2. Is the manuscript technically sound, and do the data support the conclusions?

The manuscript must describe a technically sound piece of scientific research with data that supports the conclusions. Experiments must have been conducted rigorously, with appropriate controls, replication, and sample sizes. The conclusions must be drawn appropriately based on the data presented.

Reviewer #1: Yes

Reviewer #2: Partly

**********

3. Has the statistical analysis been performed appropriately and rigorously?

Reviewer #1: Yes

Reviewer #2: No

**********

4. Have the authors made all data underlying the findings in their manuscript fully available?

The PLOS Data policy requires authors to make all data underlying the findings described in their manuscript fully available without restriction, with rare exception (please refer to the Data Availability Statement in the manuscript PDF file). The data should be provided as part of the manuscript or its supporting information, or deposited to a public repository. For example, in addition to summary statistics, the data points behind means, medians and variance measures should be available. If there are restrictions on publicly sharing data—e.g. participant privacy or use of data from a third party—those must be specified.

Reviewer #1: Yes

Reviewer #2: Yes

**********

5. Is the manuscript presented in an intelligible fashion and written in standard English?

PLOS ONE does not copyedit accepted manuscripts, so the language in submitted articles must be clear, correct, and unambiguous. Any typographical or grammatical errors should be corrected at revision, so please note any specific errors here.

Reviewer #1: Yes

Reviewer #2: No

**********

6. Review Comments to the Author

Please use the space provided to explain your answers to the questions above. You may also include additional comments for the author, including concerns about dual publication, research ethics, or publication ethics. (Please upload your review as an attachment if it exceeds 20,000 characters)

Reviewer #1: The authors have addressed the necessary advice and corrections. However, a few aspects still require improvement.

Lines 161–165: Please rewrite this section to avoid duplication.

Below Table 3: Please include the significant q values in addition to the p values.

Reviewer #2: This manuscript addresses an under-studied topic: behavioural and emotional outcomes among adolescents with congenital colour vision deficiency (CCVD) and the role of socioeconomic context. The general design (school screening with diagnostic confirmation, matched controls, and CBCL-based outcomes) is potentially suitable for PLOS ONE. However, the current revision cannot be evaluated properly due to major presentation issues (tracked-changes artefacts) and several internal inconsistencies in reporting and terminology.

**********

7. PLOS authors have the option to publish the peer review history of their article (what does this mean?). If published, this will include your full peer review and any attached files.

If you choose “no”, your identity will remain anonymous but your review may still be made public.

**Do you want your identity to be public for this peer review?**  For information about this choice, including consent withdrawal, please see our Privacy Policy.

Reviewer #1: **Yes:** Syeda Humaida Hasan

Reviewer #2: No

**********

[NOTE: If reviewer comments were submitted as an attachment file, they will be attached to this email and accessible via the submission site. Please log into your account, locate the manuscript record, and check for the action link "View Attachments". If this link does not appear, there are no attachment files.]

To ensure your figures meet our technical requirements, please review our figure guidelines: https://journals.plos.org/plosone/s/figures

You may also use PLOS’s free figure tool, NAAS, to help you prepare publication quality figures: https://journals.plos.org/plosone/s/figures#loc-tools-for-figure-preparation.

NAAS will assess whether your figures meet our technical requirements by comparing each figure against our figure specifications.

Attachment Submitted filename: CCVD.PLOS.docx

Attachment Submitted filename: Recommendation.docx

# Author comment: Author response to Decision Letter 2

**DOI:** 10.1371/journal.pone.0353847.r004

16 Feb 2026

Reviewer #1 mentioned that we have addressed all the necessary advice and corrections. We thank Reviewer #1 for the positive feedback.

For Reviewer #2, below is our response to each point raised:

Point #1:The study question is clear and the overall approach (screening-confirmed CCVD, matched comparison group, CBCL outcomes, and subgroup socioeconomic analyses) is reasonable. However, the current submission contains substantial presentation problems (tracked-changes artefacts) and several internal inconsistencies that need correction before the technical merits can be fully assessed (see detailed comments).

Our response:

In the revised submission, all tracked changes, highlights, and formatting artefacts have been fully removed, and the manuscript has been carefully proofread to ensure internal consistency across the Abstract, Methods, Results, and Discussion sections. We have also addressed all specific inconsistencies highlighted in the detailed reviewer comments.

Point #2:The use of Shapiro–Wilk for small samples and nonparametric group comparisons is appropriate, and the manuscript states that multiple-comparison adjustment was performed using Benjamini–Hochberg FDR with inference based on q-values. However, reporting is inconsistent and sometimes conflates p-values and q-values (e.g., “all qp<0.05” and “q<0.05p<0.05”), and the effect-size/CI reporting claim in Methods is duplicated and appears not to be implemented consistently across all relevant tables.

Our response:

We have revised the manuscript to ensure that statistical inference is consistently based on FDR-adjusted q-values, as specified in the Statistical Analysis section (lines 150-153). All instances of ambiguous or conflated reporting have been corrected, and statistical significance has been defined uniformly as q < 0.05.

In addition, we have reviewed and corrected the reporting of effect sizes and confidence intervals to ensure alignment with the Methods description (lines 143-145). Effect sizes are now consistently reported for all chi-square analyses using Cramér’s V, along with 95% confidence intervals for Cramér’s V estimated using nonparametric bootstrapping (1,000 resamples, percentile method).

Point #3: The submission includes a Figshare repository link for the underlying data. Before acceptance, please ensure that the repository contains the de-identified, individual-level dataset underlying all analyses (e.g., CBCL scale scores/T-scores or raw-to-T transformation inputs as applicable, group indicators, and socioeconomic variables), plus a clear data dictionary/codebook and any analysis syntax needed to reproduce results.

Our response:

The dataset in the Figshare repository has been updated. All individuals are anonymous. All data are presented with headings that include the raw score, the T-score, and the socioeconomic variables. There is no missing data.

Point #4: The file appears to contain tracked-changes/Word formatting artefacts embedded in the PDF (e.g., “Formatted: Highlight / Revised Manuscript with Track Changes”), including in the Abstract. There are also visible typographical issues (e.g., “dan and”; spacing/duplication; stray characters). A clean, publication-ready version (without tracked changes and without Word “Formatted:” tags) is required.

Our response:

All tracked changes, highlights, and formatting artefacts have been fully removed.

Point #5: Study design terminology is inconsistent and must be corrected.

In the response material, the authors state the study was not designed as a case–control study because participants were selected by exposure status, yet elsewhere it is described as a “cross-sectional case–control study”. Action needed: use a single, technically accurate description throughout (e.g., “matched cross-sectional comparative study” or equivalent) and ensure consistent use across manuscript and response text.

Our response:

We have revised the manuscript to use a single, technically accurate description throughout. The study is now consistently described as a matched cross-sectional comparative study (line 39), as participants were identified through school-based colour vision screening and compared by CCVD exposure status rather than selected based on behavioural outcomes. All references to “case–control” design have been removed from the Abstract, Methods, Results, Discussion, and response materials to ensure consistency and clarity.

Point #6: Limitations section has numbering/duplication issues (e.g., “Second, Third”); please correct structure and remove repeated phrases.

Our response:

We have corrected the structure and removed repeated phrases in the Limitation section.

Point #7: Power/sample size reporting: the accompanying review notes that power analysis assumptions are not fully specified. If a priori power was used, report assumed effect size, alpha (and whether alpha/q was targeted), and allocation. If not, describe justification and interpret results cautiously.

Our response:

The sample size was determined a priori based on the expected prevalence of CCVD among male schoolchildren and the feasibility of school-based screening. A minimum of 40 adolescents per group was targeted, assuming a large effect size (Cohen’s w = 0.6), an alpha level of 0.05, and 1:1 group allocation. This sample provides approximately 80% power to detect group differences using Chi-square tests. An additional 10% was added to account for potential non-response or incomplete data, resulting in a final target of 44 participants per group. These can be found in the revised manuscript (lines 52-57).

We also note that adjustment for multiple comparisons using the Benjamini–Hochberg FDR procedure was applied during analysis and was not incorporated into the a priori sample size estimation. Accordingly, results should be interpreted cautiously as exploratory and hypothesis-generating.

Point #8: Lines 161–165: Please rewrite this section to avoid duplication.

Our response:

Lines 161–165 have been rewritten and consolidated into a single sentence to improve clarity and avoid repetition.

Replaced at Line 163-165:

All identified adolescents with CCVD and age-matched peers with normal colour vision were aged 13–16 years (mean age = 14.52 ± 1.30 years), enrolled in Forms 1–4 of secondary school, and were of Malay ethnicity

Point #9: Below Table 3: Please include the significant q values in addition to the p values.

Our response: As suggested, the footnote to Table 3 has been revised to clarify that statistical significance was determined using q < 0.05.

Replaced Line 215-216:

*Both unadjusted p-values and FDR-adjusted q-values are reported; statistical significance was defined as q < 0.05.

Attachment Submitted filename: Rev.2_Authors Reply_PlosOne_4Feb.docx

# Peer review: Decision Letter 2

**DOI:** 10.1371/journal.pone.0353847.r005

**Authors:** Thiago Fernandes

24 Feb 2026

<div>PONE-D-25-42304R2

Behavioural and Emotional Assessment of Malaysian Adolescent Boys with Congenital Colour Vision Deficiency and its Association with Socioeconomic Factors.

PLOS One

Dear Dr. Hairol,

Thank you for submitting your manuscript to PLOS ONE. After careful consideration, we feel that it has merit but does not fully meet PLOS ONE’s publication criteria as it currently stands. Therefore, we invite you to submit a revised version of the manuscript that addresses the points raised during the review process.

Please submit your revised manuscript by Apr 10 2026 11:59PM. If you will need more time than this to complete your revisions, please reply to this message or contact the journal office at plosone@plos.org. When you're ready to submit your revision, log on to https://www.editorialmanager.com/pone/ and select the 'Submissions Needing Revision' folder to locate your manuscript file.

Please include the following items when submitting your revised manuscript:

- A letter that responds to each point raised by the academic editor and reviewer(s). You should upload this letter as a separate file labeled 'Response to Reviewers'.
- A marked-up copy of your manuscript that highlights changes made to the original version. You should upload this as a separate file labeled 'Revised Manuscript with Track Changes'.
- An unmarked version of your revised paper without tracked changes. You should upload this as a separate file labeled 'Manuscript'.

If you would like to make changes to your financial disclosure, please include your updated statement in your cover letter. Guidelines for resubmitting your figure files are available below the reviewer comments at the end of this letter.

If applicable, we recommend that you deposit your laboratory protocols in protocols.io to enhance the reproducibility of your results. Protocols.io assigns your protocol its own identifier (DOI) so that it can be cited independently in the future. For instructions see: https://journals.plos.org/plosone/s/submission-guidelines#loc-laboratory-protocols. Additionally, PLOS ONE offers an option for publishing peer-reviewed Lab Protocol articles, which describe protocols hosted on protocols.io. Read more information on sharing protocols at https://plos.org/protocols?utm_medium=editorial-email&utm_source=authorletters&utm_campaign=protocols.

We look forward to receiving your revised manuscript.

Kind regards,

Thiago P. Fernandes, PhD

Academic Editor

PLOS One

Journal Requirements:

1. If the reviewer comments include a recommendation to cite specific previously published works, please review and evaluate these publications to determine whether they are relevant and should be cited. There is no requirement to cite these works unless the editor has indicated otherwise.

Additional Editor Comments:

Please respond to all comments and highlight the changes in the revised ms.

[Note: HTML markup is below. Please do not edit.]

Reviewer's Responses to Questions

**Comments to the Author**

1. If the authors have adequately addressed your comments raised in a previous round of review and you feel that this manuscript is now acceptable for publication, you may indicate that here to bypass the “Comments to the Author” section, enter your conflict of interest statement in the “Confidential to Editor” section, and submit your "Accept" recommendation.

Reviewer #2: All comments have been addressed

**********

2. Is the manuscript technically sound, and do the data support the conclusions?

The manuscript must describe a technically sound piece of scientific research with data that supports the conclusions. Experiments must have been conducted rigorously, with appropriate controls, replication, and sample sizes. The conclusions must be drawn appropriately based on the data presented.

Reviewer #2: Partly

**********

3. Has the statistical analysis been performed appropriately and rigorously?

Reviewer #2: Yes

**********

4. Have the authors made all data underlying the findings in their manuscript fully available?

The PLOS Data policy requires authors to make all data underlying the findings described in their manuscript fully available without restriction, with rare exception (please refer to the Data Availability Statement in the manuscript PDF file). The data should be provided as part of the manuscript or its supporting information, or deposited to a public repository. For example, in addition to summary statistics, the data points behind means, medians and variance measures should be available. If there are restrictions on publicly sharing data—e.g. participant privacy or use of data from a third party—those must be specified.

Reviewer #2: Yes

**********

5. Is the manuscript presented in an intelligible fashion and written in standard English?

PLOS ONE does not copyedit accepted manuscripts, so the language in submitted articles must be clear, correct, and unambiguous. Any typographical or grammatical errors should be corrected at revision, so please note any specific errors here.

Reviewer #2: No

**********

6. Review Comments to the Author

Please use the space provided to explain your answers to the questions above. You may also include additional comments for the author, including concerns about dual publication, research ethics, or publication ethics. (Please upload your review as an attachment if it exceeds 20,000 characters)

Reviewer #2: My responses to the review questions are based on the points below.

Technical soundness and support for conclusions. The study design and measurement tools are generally suitable, and the primary comparisons between CCVD and NCV groups appear consistent with the results presented. However, several reporting and presentation issues reduce confidence in the accuracy of the statistical reporting and prevent the manuscript from being considered publication ready at this stage.

Statistical analysis. The choice of non parametric testing for group comparisons after assessing distributional assumptions is reasonable. The stated plan to control multiplicity using Benjamini to Hochberg false discovery rate is also appropriate, and the tables include both p values and q values. That said, there are inconsistencies that require correction before the reader can interpret results reliably. In particular, the manuscript reports Shapiro Wilk normality findings using q values. Normality tests produce p values, and it is not clear why FDR adjustment would be applied to normality testing. Please correct this to p values, or clearly justify and document any adjustment if it was applied. In addition, subgroup chi square analyses described as within the CCVD group repeatedly report N equal to 88 even though the CCVD group size is 44. Please correct all subgroup chi square statements to the correct N for the subgroup being analysed, and confirm that the analyses were run on the intended subset.

Data availability. A Figshare DOI is provided, which is positive and aligns with the journal’s data policy. Before acceptance, please ensure the repository includes the underlying individual level dataset required to reproduce all tables, plus a clear codebook describing variables, coding, and scoring, and sufficient information to reproduce the analyses, including how CBCL scores were handled and transformed where applicable.

Presentation and language. The main barrier to intelligibility is the manuscript file itself. The PDF includes duplicated content and material that appears to be tracked changes embedded within the text. This makes the document difficult to read and evaluate as a clean revised version. Please upload a single clean manuscript file with all track change artefacts removed.

Additional suggestions to strengthen clarity and interpretation.

a. Maintain consistent language throughout the Abstract, Results narrative, and tables regarding which values are used for inference. If you state that conclusions are based on q values, ensure the narrative highlights adjusted results consistently and avoids mixing p based and q based interpretations.

b. Because the CCVD subgroup analyses involve small cell counts and multiple comparisons, please frame these findings explicitly as exploratory, and consider reporting effect sizes or risk differences alongside significance testing to support practical interpretation.

Ethics and publication ethics. Ethical approvals are stated. I did not identify clear concerns about research ethics from the information provided. If any part of the dataset cannot be shared publicly due to privacy restrictions, this should be stated explicitly in the Data Availability section, along with the mechanism for controlled access.

In summary, the study appears potentially publishable on technical grounds, but I recommend further revision focused on correcting statistical reporting inconsistencies, ensuring accurate subgroup sample size reporting, providing a clean readable manuscript file, and strengthening reproducibility documentation in the data repository.

**********

7. PLOS authors have the option to publish the peer review history of their article (what does this mean?). If published, this will include your full peer review and any attached files.

If you choose “no”, your identity will remain anonymous but your review may still be made public.

**Do you want your identity to be public for this peer review?**  For information about this choice, including consent withdrawal, please see our Privacy Policy.

Reviewer #2: No

**********

[NOTE: If reviewer comments were submitted as an attachment file, they will be attached to this email and accessible via the submission site. Please log into your account, locate the manuscript record, and check for the action link "View Attachments". If this link does not appear, there are no attachment files.]

To ensure your figures meet our technical requirements, please review our figure guidelines: https://journals.plos.org/plosone/s/figures

You may also use PLOS’s free figure tool, NAAS, to help you prepare publication quality figures: https://journals.plos.org/plosone/s/figures#loc-tools-for-figure-preparation.

NAAS will assess whether your figures meet our technical requirements by comparing each figure against our figure specifications.

Attachment Submitted filename: Reviewed-190226.docx

# Author comment: Author response to Decision Letter 3

**DOI:** 10.1371/journal.pone.0353847.r006

2 Apr 2026

All responses have been addressed in a separate document uploaded with the revised manuscript.

The points that we would like to highlight:

1) The Data Availability statement was updated to confirm that the Figshare repository includes the dataset, codebook, and CBCL scoring documentation. A separate, detailed codebook and documentation describing CBCL score categorisation have also been uploaded to figshare. The codebook is also included in a separate tab within the dataset spreadsheet.

2) With regards to pdf containing duplicated content and tracked changes as mentioned by Reviewer #2, As per PLOS One’s requirements, for the previous submission of the manuscript, we have uploaded both the clean, revised version and the version with tracked changes to the submission system. The PDF that the PLOS One system produced thus contains the clean version followed by the tracked changes version.

We agree that the tracked changes version contains a lot of duplicated text, indicating the changes we made following the prior rounds of revision.

We have now uploaded the tracked changes version without the duplicated text, making it easier for Reviewer #2 to read and evaluate the changes we made. At the same time, several tracked changes remain to fulfil PLOS One requirements that a version with tracked changes be included in the submission.

Attachment Submitted filename: Rev.3_Authors Reply_PlosOne_April_v2.docx

# Peer review: Decision Letter 3

**DOI:** 10.1371/journal.pone.0353847.r007

**Authors:** Thiago Fernandes

14 Apr 2026

PONE-D-25-42304R3

Behavioural and Emotional Assessment of Malaysian Adolescent Boys with Congenital Colour Vision Deficiency and its Association with Socioeconomic Factors

PLOS One

Dear Dr. Hairol,

Thank you for submitting your manuscript to PLOS ONE. After careful consideration, we feel that it has merit but does not fully meet PLOS ONE’s publication criteria as it currently stands. Therefore, we invite you to submit a revised version of the manuscript that addresses the points raised during the review process.

Please respond all comments and highlight the changes in the revised ms.

I am sorry for the lengthy rounds, but although interesting the study is, the remaining reviewer raised important points.

Please submit your revised manuscript by May 29 2026 11:59PM. If you will need more time than this to complete your revisions, please reply to this message or contact the journal office at plosone@plos.org. When you're ready to submit your revision, log on to https://www.editorialmanager.com/pone/ and select the 'Submissions Needing Revision' folder to locate your manuscript file.

Please include the following items when submitting your revised manuscript:

- A letter that responds to each point raised by the academic editor and reviewer(s). You should upload this letter as a separate file labeled 'Response to Reviewers'.
- A marked-up copy of your manuscript that highlights changes made to the original version. You should upload this as a separate file labeled 'Revised Manuscript with Track Changes'.
- An unmarked version of your revised paper without tracked changes. You should upload this as a separate file labeled 'Manuscript'.

If you would like to make changes to your financial disclosure, please include your updated statement in your cover letter. Guidelines for resubmitting your figure files are available below the reviewer comments at the end of this letter.

If applicable, we recommend that you deposit your laboratory protocols in protocols.io to enhance the reproducibility of your results. Protocols.io assigns your protocol its own identifier (DOI) so that it can be cited independently in the future. For instructions see: https://journals.plos.org/plosone/s/submission-guidelines#loc-laboratory-protocols. Additionally, PLOS ONE offers an option for publishing peer-reviewed Lab Protocol articles, which describe protocols hosted on protocols.io. Read more information on sharing protocols at https://plos.org/protocols?utm_medium=editorial-email&utm_source=authorletters&utm_campaign=protocols.

As the corresponding author, your ORCID iD is verified in the submission system and will appear in the published article. PLOS supports the use of ORCID, and we encourage all coauthors to register for an ORCID iD and use it as well. Please encourage your coauthors to verify their ORCID iD within the submission system before final acceptance, as unverified ORCID iDs will not appear in the published article. *Only*  the individual author can complete the verification step; PLOS staff *cannot*  verify ORCID iDs on behalf of authors.

We look forward to receiving your revised manuscript.

Kind regards,

Thiago P. Fernandes, PhD

Academic Editor

PLOS One

Journal Requirements:

If the reviewer comments include a recommendation to cite specific previously published works, please review and evaluate these publications to determine whether they are relevant and should be cited. There is no requirement to cite these works unless the editor has indicated otherwise.

Please review your reference list to ensure that it is complete and correct. If you have cited papers that have been retracted, please include the rationale for doing so in the manuscript text, or remove these references and replace them with relevant current references. Any changes to the reference list should be mentioned in the rebuttal letter that accompanies your revised manuscript. If you need to cite a retracted article, indicate the article’s retracted status in the References list and also include a citation and full reference for the retraction notice.

[Note: HTML markup is below. Please do not edit.]

Reviewers' comments:

Reviewer's Responses to Questions

**Comments to the Author**

1. If the authors have adequately addressed your comments raised in a previous round of review and you feel that this manuscript is now acceptable for publication, you may indicate that here to bypass the “Comments to the Author” section, enter your conflict of interest statement in the “Confidential to Editor” section, and submit your "Accept" recommendation.

Reviewer #2: (No Response)

**********

2. Is the manuscript technically sound, and do the data support the conclusions?

The manuscript must describe a technically sound piece of scientific research with data that supports the conclusions. Experiments must have been conducted rigorously, with appropriate controls, replication, and sample sizes. The conclusions must be drawn appropriately based on the data presented.

Reviewer #2: No

**********

3. Has the statistical analysis been performed appropriately and rigorously?

Reviewer #2: N/A

**********

4. Have the authors made all data underlying the findings in their manuscript fully available?

The PLOS Data policy requires authors to make all data underlying the findings described in their manuscript fully available without restriction, with rare exception (please refer to the Data Availability Statement in the manuscript PDF file). The data should be provided as part of the manuscript or its supporting information, or deposited to a public repository. For example, in addition to summary statistics, the data points behind means, medians and variance measures should be available. If there are restrictions on publicly sharing data—e.g. participant privacy or use of data from a third party—those must be specified.

Reviewer #2: Yes

**********

5. Is the manuscript presented in an intelligible fashion and written in standard English?

PLOS ONE does not copyedit accepted manuscripts, so the language in submitted articles must be clear, correct, and unambiguous. Any typographical or grammatical errors should be corrected at revision, so please note any specific errors here.

Reviewer #2: Yes

**********

6. Review Comments to the Author

Please use the space provided to explain your answers to the questions above. You may also include additional comments for the author, including concerns about dual publication, research ethics, or publication ethics. (Please upload your review as an attachment if it exceeds 20,000 characters)

Reviewer #2: Thank you for the revision. The study addresses an underexplored and potentially important topic, and the revised version has improved in several respects. The design remains appropriate for an observational school-based comparative study, the colour vision assessment is clearly described, the CBCL/4–18 procedures and thresholds are reported, and the authors state that all underlying data, codebook, and scoring documentation are now available in Figshare via DOI.

However, under PLOS ONE’s criteria, conclusions must be supported by the data and the analyses must be described to a high technical standard. The current version still contains internal inconsistencies substantial enough to prevent acceptance in its present form. The reviewer guidelines explicitly emphasize that experiments, statistics, and other analyses must be performed to a high technical standard, that conclusions must be supported by the data, and that the article must be intelligible and written in standard English.

My main concern is that the narrative interpretation of the socioeconomic subgroup analyses does not appear to match the tabulated data. In the Results text, the manuscript states that lower parental education is significantly related to worse behavioural outcomes among adolescents with CCVD. However, in Table 5, the broad-band frequencies shown for Externalizing Problems and Total Problems appear higher in the tertiary-education subgroup than in the secondary-education subgroup. The Results text on parental education, the counts in Table 5, the Discussion, the Abstract, and the Conclusion therefore do not seem fully aligned. This issue must be checked carefully against the source dataset and corrected throughout the manuscript.

A related issue affects the household-income interpretation. Table 6 supports significant associations for Withdrawn, Anxious/Depressed, Thought Problems, and Aggressive Behaviour, but not for Attention Problems, Externalizing Problems, or Total Problems after FDR correction. Despite this, the Conclusion states that low household income is significantly associated with higher scores in domains such as Attention Problems and Externalizing Problems. That statement should be revised so that it matches the reported q values exactly.

There is also an inconsistency between the abstract and the main Results regarding the broad-band findings. In the Results section and Table 3, both Internalizing Problems and Externalizing Problems are reported as significantly higher in the CCVD group. In the abstract, however, only Externalizing Problems is highlighted at the broad-band level. Please harmonize the abstract, Results, Discussion, and Conclusion.

On statistical reporting, the general analytical approach is reasonable. The manuscript describes normality assessment with Shapiro-Wilk, non-parametric group comparisons with Mann-Whitney U, chi-square tests for categorized T-scores, and FDR adjustment with inference based on q values. Still, because the subgroup findings are central to the paper’s interpretation, the authors should re-check the subgroup tables and all corresponding narrative statements carefully before any final decision.

On data availability, the revision is improved and appears consistent with the journal’s requirements. The manuscript now states that the dataset, codebook, and scoring documentation are available in Figshare through a DOI, which supports reproducibility.

The manuscript is generally readable, but a few language and presentation issues remain. For example, “The CBCLwere distributed” should be corrected, and several sentences need tightening to ensure exact agreement between the tables and the narrative text.

In summary, this manuscript has merit and may become suitable for publication, but it is not yet acceptable in its current form because the principal socioeconomic conclusions are not fully secure as written. Please verify Tables 5 and 6 against the source data, correct all affected interpretations in the Abstract, Results, Discussion, and Conclusion, and ensure that the final claims are strictly limited to the results actually supported by the reported analyses.

**********

7. PLOS authors have the option to publish the peer review history of their article (what does this mean?). If published, this will include your full peer review and any attached files.

If you choose “no”, your identity will remain anonymous but your review may still be made public.

**Do you want your identity to be public for this peer review?**  For information about this choice, including consent withdrawal, please see our Privacy Policy.

Reviewer #2: No

**********

[NOTE: If reviewer comments were submitted as an attachment file, they will be attached to this email and accessible via the submission site. Please log into your account, locate the manuscript record, and check for the action link "View Attachments". If this link does not appear, there are no attachment files.]

To ensure your figures meet our technical requirements, please review our figure guidelines: https://journals.plos.org/plosone/s/figures

You may also use PLOS’s free figure tool, NAAS, to help you prepare publication quality figures: https://journals.plos.org/plosone/s/figures#loc-tools-for-figure-preparation.

NAAS will assess whether your figures meet our technical requirements by comparing each figure against our figure specifications.

Attachment Submitted filename: Reviewer report for authors120426.docx

# Author comment: Author response to Decision Letter 4

**DOI:** 10.1371/journal.pone.0353847.r008

26 May 2026

A separate response to reviewer file has been uploaded in the submission system.

Attachment Submitted filename: Response_to_Reviewers_auresp_4.docx

# Editor report: Decision Letter 4

**DOI:** 10.1371/journal.pone.0353847.r009

**Authors:** Thiago Fernandes

18 Jun 2026

PONE-D-25-42304R4

Behavioural and Emotional Assessment of Malaysian Adolescent Boys with Congenital Colour Vision Deficiency and its Association with Socioeconomic Factors

PLOS One

Dear Dr. Hairol,

Thank you for submitting your manuscript to PLOS ONE. After careful consideration, we feel that it has merit but does not fully meet PLOS ONE’s publication criteria as it currently stands. Therefore, we invite you to submit a revised version of the manuscript that addresses the points raised during the review process.

==============================

Thank you for your thoughtful and careful edits. I appreciate the substantial effort invested in addressing the reviewers comments, and I am pleased to see that nearly all concerns have been satisfactorily resolved. The revised ms is considerably improved in terms of consistency, interpretation of the subgroup analyses, and overall presentation of the findings. To ensure the accuracy and consistency of the revised ms, I would kindly ask the authors to address the following points. 1. Please verify the confidence interval reported for Cramér's V in Table 5 (Externalising Problems), as the reported interval appears inconsistent and may contain a typographical error. 2. Please review the Discussion and Conclusion sections to ensure that all interpretations remain strictly associative rather than causal, given the cross-sectional design of the study. 3. Please perform a final consistency check between the tables and the corresponding narrative descriptions throughout the ms. 4. Please verify that all reported confidence intervals, effect sizes, and statistical values are presented accurately and consistently. 5. Please conduct a final proofreading review to correct any remaining typographical, formatting, or presentation issues. I look forward to receiving the revised version.

==============================

Please submit your revised manuscript by Aug 02 2026 11:59PM. If you will need more time than this to complete your revisions, please reply to this message or contact the journal office at plosone@plos.org. When you're ready to submit your revision, log on to https://www.editorialmanager.com/pone/ and select the 'Submissions Needing Revision' folder to locate your manuscript file.

Please include the following items when submitting your revised manuscript:

- A letter that responds to each point raised by the academic editor and reviewer(s). You should upload this letter as a separate file labeled 'Response to Reviewers'.
- A marked-up copy of your manuscript that highlights changes made to the original version. You should upload this as a separate file labeled 'Revised Manuscript with Track Changes'.
- An unmarked version of your revised paper without tracked changes. You should upload this as a separate file labeled 'Manuscript'.

If you would like to make changes to your financial disclosure, please include your updated statement in your cover letter. Guidelines for resubmitting your figure files are available below the reviewer comments at the end of this letter.

If applicable, we recommend that you deposit your laboratory protocols in protocols.io to enhance the reproducibility of your results. Protocols.io assigns your protocol its own identifier (DOI) so that it can be cited independently in the future. For instructions see: https://journals.plos.org/plosone/s/submission-guidelines#loc-laboratory-protocols. Additionally, PLOS ONE offers an option for publishing peer-reviewed Lab Protocol articles, which describe protocols hosted on protocols.io. Read more information on sharing protocols at https://plos.org/protocols?utm_medium=editorial-email&utm_source=authorletters&utm_campaign=protocols.

As the corresponding author, your ORCID iD is verified in the submission system and will appear in the published article. PLOS supports the use of ORCID, and we encourage all coauthors to register for an ORCID iD and use it as well. Please encourage your coauthors to verify their ORCID iD within the submission system before final acceptance, as unverified ORCID iDs will not appear in the published article. *Only*  the individual author can complete the verification step; PLOS staff *cannot*  verify ORCID iDs on behalf of authors.

We look forward to receiving your revised manuscript.

Kind regards,

Thiago P. Fernandes, PhD

Academic Editor

PLOS One

Journal Requirements:

If the reviewer comments include a recommendation to cite specific previously published works, please review and evaluate these publications to determine whether they are relevant and should be cited. There is no requirement to cite these works unless the editor has indicated otherwise.

Please review your reference list to ensure that it is complete and correct. If you have cited papers that have been retracted, please include the rationale for doing so in the manuscript text, or remove these references and replace them with relevant current references. Any changes to the reference list should be mentioned in the rebuttal letter that accompanies your revised manuscript. If you need to cite a retracted article, indicate the article’s retracted status in the References list and also include a citation and full reference for the retraction notice.

[Note: HTML markup is below. Please do not edit.]

[NOTE: If reviewer comments were submitted as an attachment file, they will be attached to this email and accessible via the submission site. Please log into your account, locate the manuscript record, and check for the action link "View Attachments". If this link does not appear, there are no attachment files.]

To ensure your figures meet our technical requirements, please review our figure guidelines: https://journals.plos.org/plosone/s/figures

You may also use PLOS’s free figure tool, NAAS, to help you prepare publication quality figures: https://journals.plos.org/plosone/s/figures#loc-tools-for-figure-preparation.

NAAS will assess whether your figures meet our technical requirements by comparing each figure against our figure specifications.

# Author comment: Author response to Decision Letter 5

**DOI:** 10.1371/journal.pone.0353847.r010

29 Jun 2026

Response to reviewers have been uploaded as a separate file. In summary:

a) We corrected a typographical error regarding the confidence interval for Cramér's V in Table 5 (Externalising Problems). We also verified that all confidence intervals, effect sizes, and p-values are formatted accurately and consistently throughout the text (e.g., ensuring leading zeros and unified χ² notation).

b) Given the cross-sectional design of our study, we meticulously reviewed the Discussion and Conclusion sections to remove any causal wording. All interpretations have been strictly rephrased to reflect associative relationships (e.g., using terms like "associated with," "linked to," and "observed in").

c) We performed a comprehensive cross-check to ensure perfect alignment between the data presented in the tables and the corresponding narrative descriptions in the manuscript.

d) A final, thorough proofreading was conducted to correct any remaining typographical errors, improve sentence clarity, fix spacing issues, and ensure consistent terminology throughout the text.

Attachment Submitted filename: Rev.5_Authors Reply_PlosOne_20June.docx

# Editor report: Decision Letter 5

**DOI:** 10.1371/journal.pone.0353847.r011

**Authors:** Thiago Fernandes

30 Jun 2026

Behavioural and Emotional Assessment of Malaysian Adolescent Boys with Congenital Colour Vision Deficiency and its Association with Socioeconomic Factors

PONE-D-25-42304R5

Dear Dr. Hairol,

We’re pleased to inform you that your manuscript has been judged scientifically suitable for publication and will be formally accepted for publication once it meets all outstanding technical requirements.

Within one week, you’ll receive an e-mail detailing the required amendments. When these have been addressed, you’ll receive a formal acceptance letter and your manuscript will be scheduled for publication.

An invoice will be generated when your article is formally accepted. Please note, if your institution has a publishing partnership with PLOS and your article meets the relevant criteria, all or part of your publication costs will be covered. Please make sure your user information is up-to-date by logging into Editorial Manager at Editorial Manager® and clicking the ‘Update My Information' link at the top of the page. For questions related to billing, please contact billing support.

If your institution or institutions have a press office, please notify them about your upcoming paper to help maximize its impact. If they’ll be preparing press materials, please inform our press team as soon as possible -- no later than 48 hours after receiving the formal acceptance. Your manuscript will remain under strict press embargo until 2 pm Eastern Time on the date of publication. For more information, please contact onepress@plos.org.

Kind regards,

Thiago P. Fernandes, PhD

Academic Editor

PLOS One

Additional Editor Comments (optional):

Thank you for your continued efforts throughout the review process. The work invested in revising the manuscript across multiple rounds has substantially improved its clarity and methodological transparency. Thank you for your careful responses to the editorial and reviewer comments. I wish you success with the study.

Reviewers' comments:

# Editor report: Acceptance letter

**DOI:** 10.1371/journal.pone.0353847.r012

**Authors:** Thiago Fernandes

PONE-D-25-42304R5

PLOS One

Dear Dr. Hairol,

I'm pleased to inform you that your manuscript has been deemed suitable for publication in PLOS One. Congratulations! Your manuscript is now being handed over to our production team.

At this stage, our production department will prepare your paper for publication. This includes ensuring the following:

* All references, tables, and figures are properly cited

* All relevant supporting information is included in the manuscript submission,

* There are no issues that prevent the paper from being properly typeset

You will receive further instructions from the production team, including instructions on how to review your proof when it is ready. Please keep in mind that we are working through a large volume of accepted articles, so please give us a few days to review your paper and let you know the next and final steps.

Lastly, if your institution or institutions have a press office, please let them know about your upcoming paper now to help maximize its impact. If they'll be preparing press materials, please inform our press team within the next 48 hours. Your manuscript will remain under strict press embargo until 2 pm Eastern Time on the date of publication. For more information, please contact onepress@plos.org.

You will receive an invoice from PLOS for your publication fee after your manuscript has reached the completed accept phase. If you receive an email requesting payment before acceptance or for any other service, this may be a phishing scheme. Learn how to identify phishing emails and protect your accounts at https://explore.plos.org/phishing.

If we can help with anything else, please email us at customercare@plos.org.

Thank you for submitting your work to PLOS ONE and supporting open access.

Kind regards,

PLOS ONE Editorial Office Staff

on behalf of

Dr. Thiago P. Fernandes

Academic Editor

PLOS One
